# Cyclochlorotine Hydroxylase CctR Reveals DUF3328 as a Family of Copper‐Dependent Metalloenzymes

**DOI:** 10.1002/anie.202512449

**Published:** 2025-08-22

**Authors:** Wentao Huang, Jakob K. Reinhardt, Anru Tian, Xiao Zhang, Binghui Li, Noah Gould, Sashirekha Nallapati, Alexander R. Ivanov, Yi Wang, Jason J. Guo, David E. Budil, Jing‐Ke Weng

**Affiliations:** ^1^ Institute for Plant‐Human Interface Northeastern University Boston MA 02115 USA; ^2^ Department of Biology Massachusetts Institute of Technology Cambridge MA 02139 USA; ^3^ Department of Chemistry and Chemical Biology Northeastern University Boston MA 02115 USA; ^4^ Department of Bioengineering Northeastern University Boston MA 02115 USA; ^5^ Whitehead Institute for Biomedical Research Cambridge MA 02142 USA; ^6^ Department of Biological Engineering Massachusetts Institute of Technology Cambridge MA 02139 USA; ^7^ Department of Chemistry Massachusetts Institute of Technology Cambridge MA 02139 USA; ^8^ Department of Physics The Chinese University of Hong Kong Hong Kong P. R. China; ^9^ Barnett Institute of Chemical and Biological Analysis Northeastern University Boston MA 02115 USA; ^10^ Department of Chemical Engineering Northeastern University Boston MA 02115 USA

**Keywords:** Enzyme catalysis, Hydroxylase, Metalloenzymes, Natural product biosynthesis, Non‐ribosomal peptides

## Abstract

DUF3328 is a protein family widely found in fungal natural product biosynthesis pathways. Although DUF3328 proteins have long been implicated in diverse modifications of inert C(*sp^3^*)─H bonds, including halogenation, hydroxylation, and macrocyclization, the biochemical properties and catalytic mechanisms of DUF3328 proteins remain elusive. Here, we report the characterization of the DUF3328 protein CctR, which catalyzes C(*sp^3^*)─H hydroxylation of fungal cyclic peptide cyclochlorotine. Through AlphaFold modeling, in vitro biochemical characterization, and spectroscopic analysis, we demonstrate that CctR is a membrane‐associated copper‐dependent enzyme that functions as a homodimer. The dimerization of CctR is mediated by its transmembrane helix, a four‐helix coiled coil, and C‐terminal disulfide bonds. The conserved HxxHC(x)_n_HxxHC motif, characteristic of the DUF3328 superfamily, is anchored on the dimerization interface and forms a binuclear copper coordination center. Moreover, we show that CctR is dioxygen‐dependent and requires electron input for the hydroxylation reaction. Together, these findings define DUF3328 as a previously unrecognized family of binuclear copper‐dependent metalloenzymes, capable of catalyzing diverse chemical transformations, and lay the groundwork for future discovery of novel biocatalysts within this widespread enzyme class.

## Graphical abstract

DUF3328 is an enigmatic enzyme family implicated in diverse transformations in fungal natural product biosynthesis, yet its functions remain largely underexplored. Through biochemical characterization of the cyclochlorotine hydroxylase CctR, we define DUF3328 as a family of copper‐dependent metalloenzymes. This study reveals key structural and mechanistic features shared across the family.

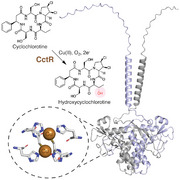

Cyclochlorotine is a non‐ribosomal cyclic peptide produced by the ascomycetous fungus *Talaromyces islandicus* (historically known as *Penicillium islandicum*)^[^ ^1^, ^2^, ^3^, ^4^ ^]^ (Figure 1). One of its derivatives, hydroxycyclochlorotine, contains a hydroxyl group on the β‐carbon of the 2‐aminobutyrate residue in the cyclic peptide. The biosynthetic gene cluster (BGC) responsible for cyclochlorotine was identified in a previous study^[^ ^1^ ^]^ and further refined based on our RNA‐seq results (Figure S1). Within this cluster, three genes—*CctO*, *CctP2*, and *CctR*—encode proteins belonging to the Domain of Unknown Function 3328 (DUF3328) family (Figure S1). Gene knockout experiments conducted by us (Figure S2) and another group^[^ ^5^ ^]^ indicate that *CctR* encodes the hydroxylase responsible for converting cyclochlorotine to hydroxycyclochlorotine. DUF3328 is a protein family widely distributed in fungal natural product biosynthetic pathways. Members of this family have long been implicated in diverse modifications of inert C(*sp^3^*)─H bonds and occasionally in other reactions, including halogenation, hydroxylation, macrocyclization, and isomerization,^[^ ^5^, ^6^, ^7^, ^8^, ^9^ ^]^ yet their biochemical properties remain poorly understood. Here, we report the biochemical characterization of CctR and reveal the structural and mechanistic features of this newly defined class of hydroxylases which catalyze C(*sp^3^*)─H hydroxylation.

**Figure 1:**
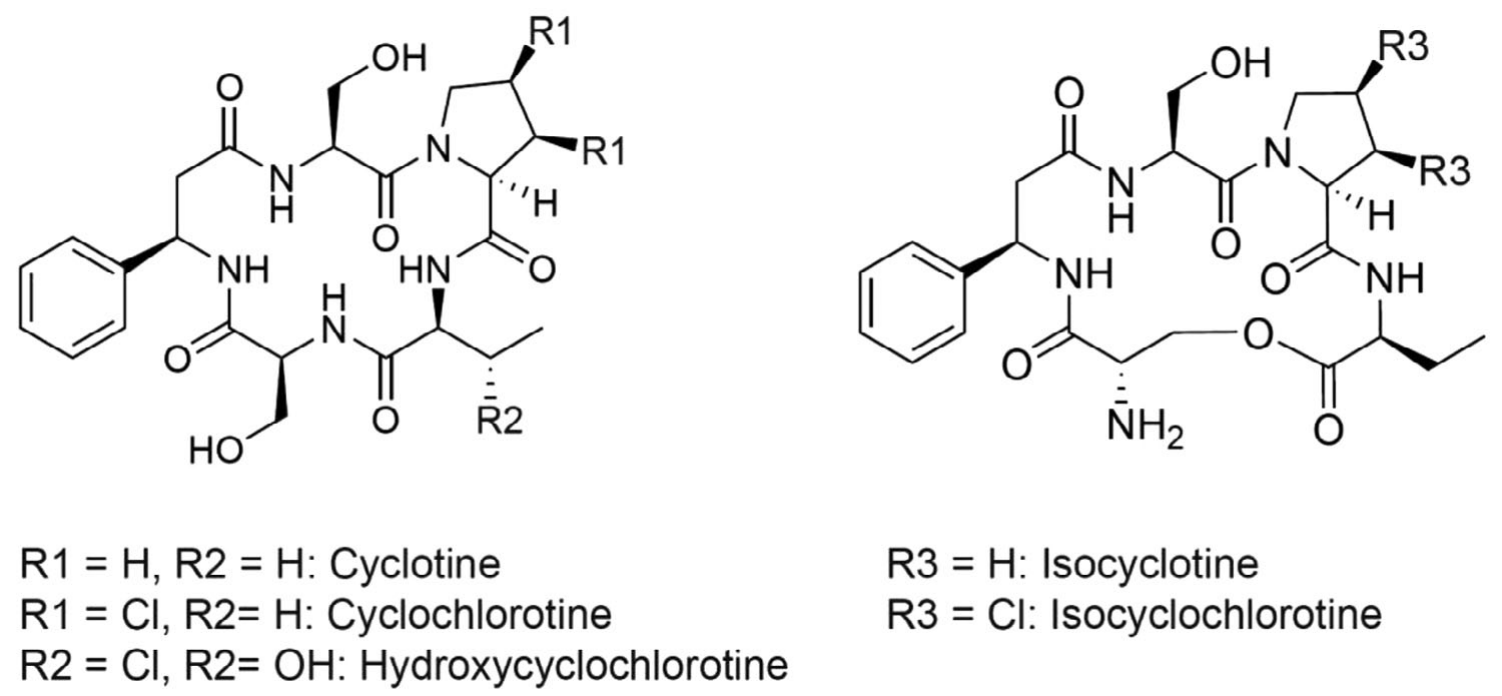
Chemical structures of cyclochlorotine and its derivatives. Shown on the left are the earliest discovered cyclochlorotine derivatives,^[^ ^1^, ^4^ ^]^ while the right side displays newly reported isomers of the cyclochlorotine derivatives.^[^ ^5^ ^]^

Using Phobius,^[^ ^10^ ^]^ a bioinformatics tool for predicting transmembrane topology and signal peptides, we analyzed the CctR protein sequence and identified a single transmembrane region spanning residues 38–59 (Figure S3). The analysis also predicted a cytoplasmic N‐terminus and a non‐cytoplasmic C‐terminus. Initial attempts to produce and purify recombinant CctR in *E. coli* BL21(DE3) strain yielded no soluble protein—an expected outcome given CctR's predicted membrane‐associated nature and *E. coli*’s lack of appropriate membrane‐bound organelles. We subsequently expressed full‐length CctR in *Spodoptera frugiperda* Sf9 cells, an insect cell line commonly used for membrane protein expression. Notably, CctR expressed in Sf9 cells predominantly appeared as a band corresponding to the size of a dimer on SDS‐PAGE in the absence of β‐mercaptoethanol (Figure S4). Upon treatment with β‐mercaptoethanol, this dimer band shifted to a monomeric form (Figure S4). This observation suggests that CctR likely forms a homodimer in vivo, with dimerization mediated by disulfide bonds rather than solely by hydrophobic interactions, which would be disrupted by SDS. This finding is consistent with the predicted non‐cytoplasmic localization of the C‐terminal region, as disulfide bonds are not maintained in the reducing environment of the cytoplasm.

The recent release of AlphaFold has propelled substantial progress in protein structural biology.^[^ ^11^, ^12^ ^]^ Given our experimental evidence that CctR likely forms a homodimer in Sf9 cells, we employed AlphaFold to predict its homodimeric structure (Figure 2). The predicted structural model reveals that each monomer consists of an N‐terminal disordered region, a transmembrane helix, and a C‐terminal helical domain (Figure 2a). A recent study showed that CctR localizes to the membrane of the Golgi, endosomes, and vacuoles.^[^ ^13^ ^]^ Integrating the results from Phobius and AlphaFold, we propose that CctR is anchored to organelle membranes via its transmembrane helix, with the N‐terminal disordered region facing the cytosol and the large C‐terminal helical domain residing within the organelle lumen. The predicted homodimer is stabilized through multiple interactions: the transmembrane helix, a four‐helix coiled coil (Figure S5a), and two inter‐monomer disulfide bonds between C190 of one monomer and C213 of the other (Figure S5b). Mutation of C190 and C213 to alanine largely abolished dimer formation (Figure S6). The model also predicts an intra‐monomer disulfide bond between C229 and C240. Most DUF3328 proteins share a conserved HxxHC(x)_n_HxxHC motif near their C‐terminus^[^ ^14^, ^15^ ^]^ (Figure S7), which in the predicted CctR structure is anchored within the four‐helix coiled‐coil region (Figure 2b). Each helix contains an HxxHC motif that pairs with the corresponding HxxHC motif from an adjacent helix within the same monomer. In the predicted structural model, the four histidine residues align in a planar arrangement, while the two cysteine residues are positioned axially beneath this plane (Figure 2c). The four‐helix coiled coil provides a structural scaffold for these histidine and cysteine residues, which partly explains the propensity of CctR monomers to form a homodimer in vivo. Notably, this planar histidine arrangement resembles that observed in recently characterized BURP domain proteins, which are copper‐dependent macrocyclases involved in plant ribosomal cyclic peptide biosynthesis.^[^ ^16^, ^17^ ^]^ BURP domains also feature four conserved histidines arranged in a plane that coordinates two copper ions at the active site. Given this structural resemblance and the well‐established role of histidine and cysteine residues in metal coordination, CctR likely functions as a metalloenzyme.

**Figure 2:**
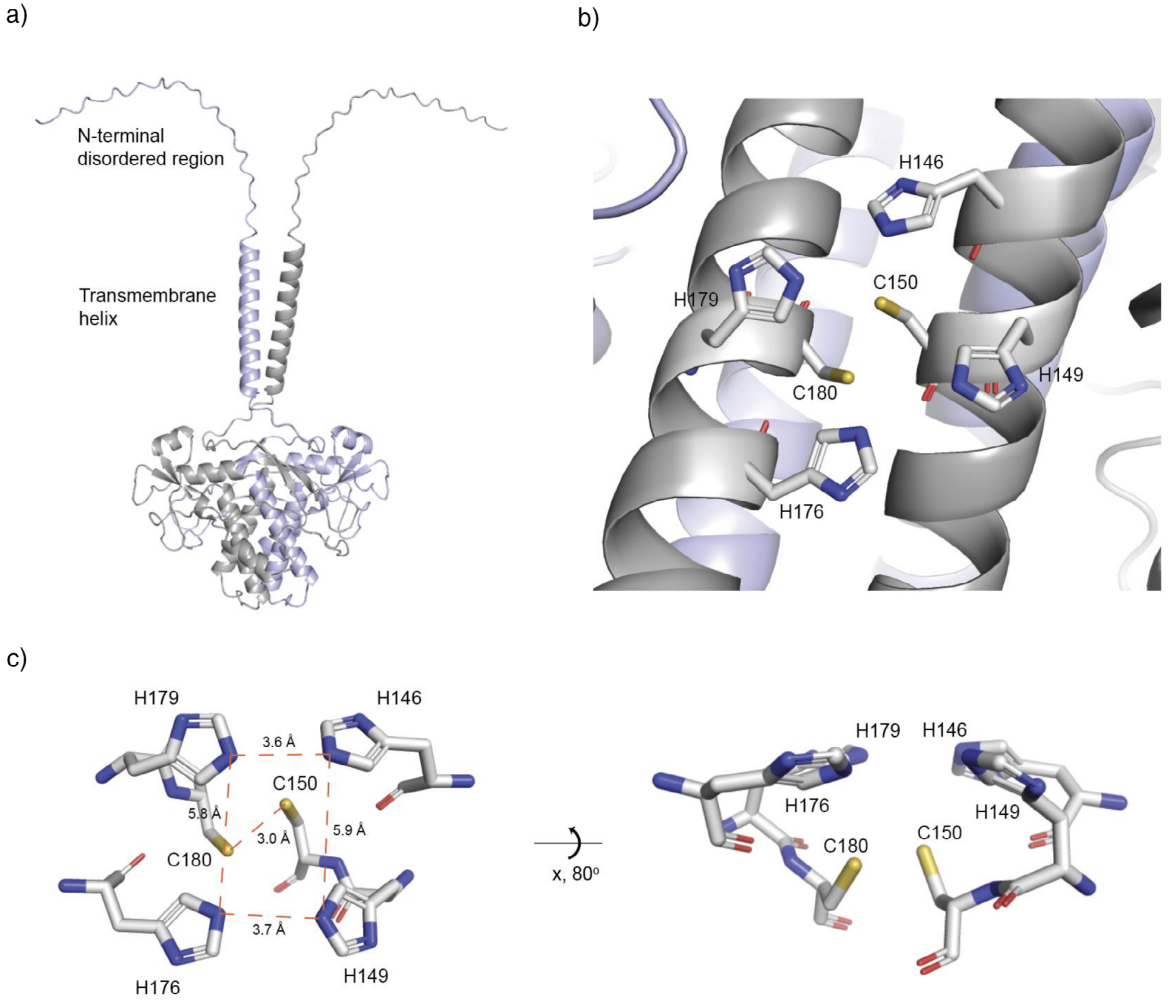
AlphaFold‐predicted structure of CctR reveals the overall architecture of the protein and the spatial organization of the conserved HxxHC(x)_n_HxxHC motif. a) AlphaFold‐predicted homodimeric structure of CctR shown in cartoon representation, with the two monomers colored in light blue and grey. b) The HxxHC(x)_n_HxxHC motif anchored within the four‐helix coiled coil at the dimerization interface. c) Left: Top view showing the spatial arrangement of the HxxHC(x)_n_HxxHC motif within one monomer, with distances between histidine Nε atoms shown in Ångstroms. Right: Side view of the HxxHC(x)_n_HxxHC motif.

Guided by the AlphaFold‐predicted structure, we proceeded with heterologous expression and purification of full‐length CctR in Sf9 cells. Purification was challenging due to the presence of the N‐terminal disordered region and the transmembrane domain. Since CctR is a membrane‐bound protein, we employed detergent n‐dodecyl‐β‐D‐maltoside (DDM) to solubilize the recombinant CctR protein during purification. The N‐terminal disordered region contributed to aggregation and nonspecific interactions with other proteins. To improve purity, we appended a twin‐strep tag to the C‐terminus of CctR. From a four liter Sf9 cell culture, we obtained approximately 0.5 mg of soluble protein at high purity. Most of the purified protein migrated as a dimer on SDS‐PAGE, which was reduced to a monomer upon treatment with β‐mercaptoethanol (Figure S8). The native oligomeric state of the purified protein was confirmed to be dimeric by Blue Native PAGE—a gel electrophoresis method optimized for membrane proteins (Figure S9) —and native protein mass spectrometry (Figure S10). Notably, when CctR was reduced by β‐mercaptoethanol, the protein appeared as high‐molecular‐weight aggregates rather than as monomers on Blue Native PAGE (Figure S8), suggesting that monomers are unstable and consistent with CctR predominantly existing as a dimer. The oxidative environment of organelle lumen likely facilitates the formation of disulfide bonds in the non‐cytoplasmic C‐terminal region of CctR, stabilizing its dimeric structure.

To evaluate the potential hydroxylase activity of purified CctR, we conducted in vitro enzymatic assays in the presence of various biologically relevant divalent metals, including Cu(II), Fe(II), Co(II), Ni(II), Mn(II), Zn(II), and Mg(II) (Figure 3a). Among these metals, only Cu(II) supported the conversion of cyclochlorotine to hydroxycyclochlorotine under aerobic conditions. This suggests the CctR enzyme purified from Sf9 cells is in its apo form and requires Cu(II) in its holo state. The copper dependency parallels that observed in recently characterized BURP domain proteins from plants.^[^ ^16^, ^17^ ^]^ Copper can participate in enzymatic reactions in either Cu(II) or Cu(I) oxidation states. Proteins are known to reduce Cu(II) to Cu(I)—a principle underlying the widely used bicinchoninic acid (BCA) assay to quantify protein concentration.^[^ ^18^ ^]^ Consistent with this, we observed the formation of Cu(I) when CctR protein was incubated with Cu(II) (Figure S11). To investigate which copper species is catalytically active in CctR, we first supplemented reactions with reducing agents (i.e., ascorbic acid, NADH, or NADPH) to promote the reduction of Cu(II) to Cu(I). Each reducing agent significantly enhanced enzymatic activity, leading to increased hydroxycyclochlorotine production (Figure 3b). Furthermore, addition of BCA—a Cu(I)‐specific chelator—completely abolished product formation (Figure 3c), demonstrating that Cu(I) is essential for the hydroxylation reaction. To ensure BCA itself did not nonspecifically inhibit enzyme function, we tested a Fe(II)/2‐oxoglutarate‐dependent oxygenase (2OGD) as a control and showed that its activity was unaffected after BCA treatment (Figure S12). The requirement for Cu(I) is consistent with mechanistic expectations for C(*sp* ^3^)─H bond activation. Analogous to cytochrome P450 (CYP) monooxygenases and 2OGDs, which employ reactive Fe(IV)═O intermediates to activate inert C(*sp* ^3^)─H bonds,^[^ ^19^, ^20^ ^]^ copper‐dependent DUF3328 proteins likely utilize the more reactive Cu(I) species rather than Cu(II). Given that Cu(I) is unstable in aqueous solution and that CctR's C‐terminus resides in the oxidative lumen of organelles, we hypothesize the involvement of an accessory protein that facilitates electron transfer or delivers Cu(I) to CctR—similar to the role of P450 reductase.

**Figure 3:**
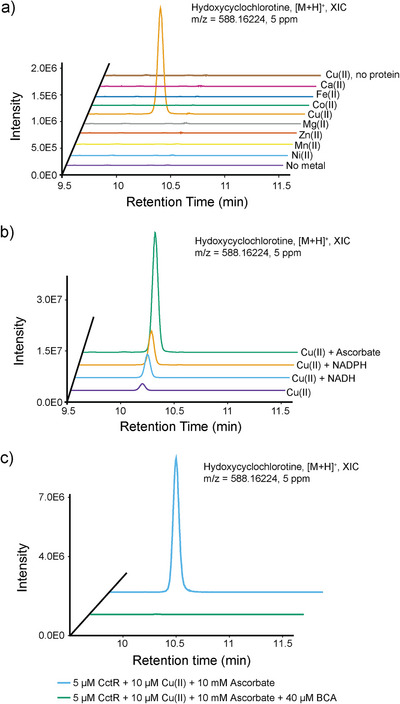
Biochemical characterization of CctR. a) Extracted ion chromatograms of hydroxycyclochlorotine produced in CctR enzymatic assays performed with different divalent metals. b) Hydroxycyclochlorotine formation in the presence of various reducing agents. The quantification of peak area is provided in Table S1. c) Effect of Cu(I)‐specific chelator BCA on CctR activity.

Based on our experimental validation of CctR's copper dependency, we employed AlphaFold3 to generate an updated homodimeric structure with bound copper (Figure S13a). The AlphaFold3 model automatically positioned the copper ions within the HxxHC(x)_n_HxxHC motif. The spatial arrangement of the four histidine residues suggests that the active site accommodates two copper ions. To determine the stoichiometry of copper binding, we analyzed the copper‐bound CctR protein using inductively coupled plasma mass spectrometry (ICP‐MS) (Figure S13b), which confirmed a 2:1 molar ratio of copper ions to CctR monomer. Continuous‐wave electron paramagnetic resonance (EPR) spectroscopy further confirmed the presence of Cu(II) bound to CctR (Figure S13c). To validate the HxxHC(x)_n_HxxHC motif as the copper‐binding site, we performed hyperfine sublevel correlation (HYSCORE) analysis of the copper‐bound CctR protein using a pulsed EPR spectrometer. The resulting spectra closely resembled those of copper bound to histidine‐rich glycoproteins^[^ ^21^ ^]^ and indicated that copper is coordinated by nitrogen atoms (Figure S13d). However, likely due to the disordered region and transmembrane domain of CctR, the resolution of the HYSCORE spectrum was limited, preventing precise determination of the number of coordinating nitrogen atoms. Accurate mapping of the EPR‐active copper center will require more detailed spectroscopic analysis of electron‐nuclear couplings in future studies. To confirm the functional importance of the HxxHC(x)_n_HxxHC motif, we generated point mutants by substituting the conserved histidine and cysteine residues with alanine. As expected, these mutants completely lost enzymatic activity (Figure S14a). Since the HxxHC(x)_n_HxxHC motif is anchored within the coiled‐coil domain at the dimerization interface, proper dimerization is essential for copper binding and enzymatic function. Consistent with this, CctR lost activity upon reduction with dithiothreitol (DTT) (Figure S14b).

To investigate whether CctR utilizes dioxygen for hydroxylation reaction, we conducted in vitro enzyme assays under an atmosphere of ^18^O_2_. Under standard air and H_2_O conditions, the mass spectrum of the produced hydroxycyclochlorotine matched the simulated natural isotopic distribution (Figure 4a,b). In contrast, assays performed under ^18^O_2_ showed a +2 *m/z* shift in the mass spectrum (Figure 4c), indicating that oxygen atom in the hydroxyl group is derived from O_2_. These results confirm that CctR is an oxygen‐dependent enzyme that utilizes O_2_ as its oxidant, similar to CYPs and 2OGDs.

**Figure 4:**
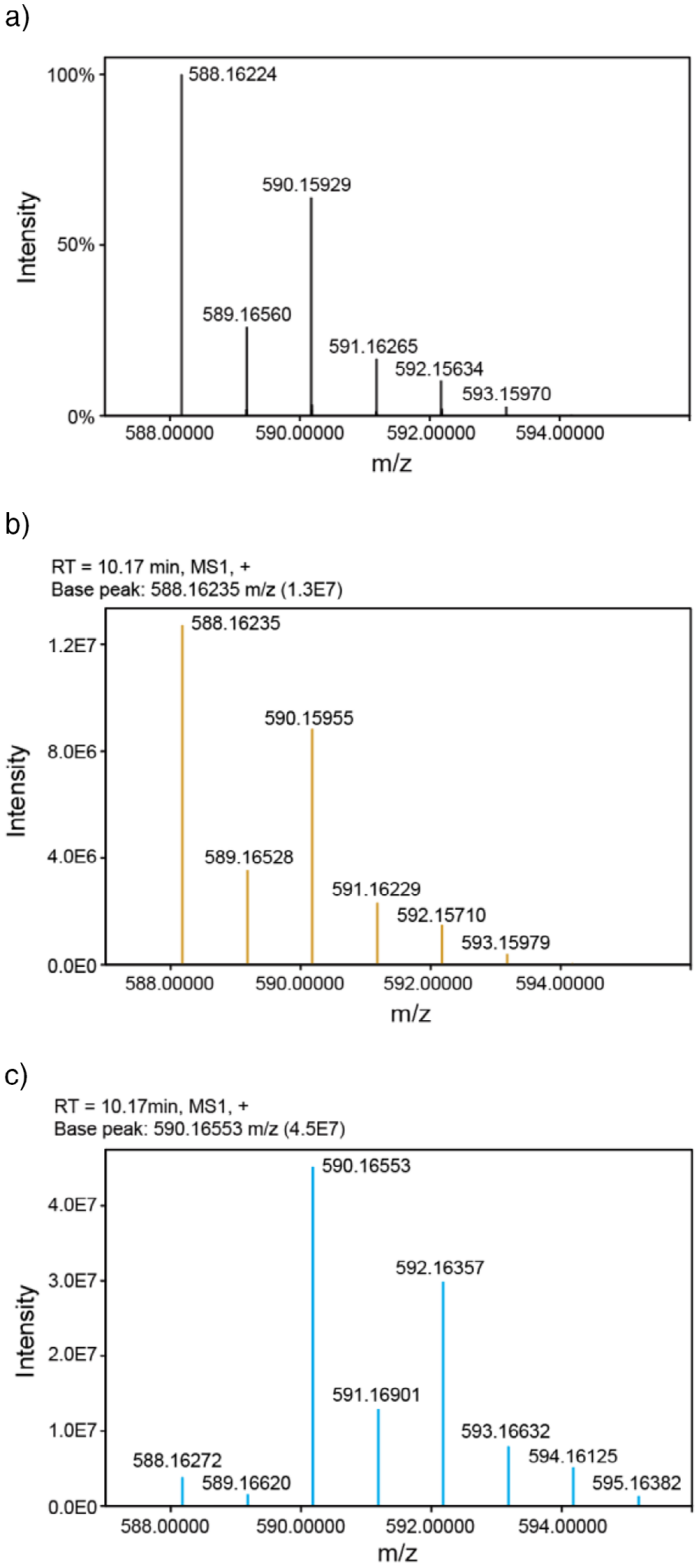
Isotope‐labeling experiments reveal that CctR utilizes dioxygen for hydroxylation reaction. a) Simulated natural isotopic distribution of hydroxycyclochlorotine (C_24_H_31_Cl_2_N_5_O_8_) [M + H]^+^. b) Mass spectrum of hydroxycyclochlorotine from a reaction conducted under ambient air. c) Mass spectrum showing a +2 *m/z* shift when the reaction was conducted under an atmosphere of ^18^O_2_.

Based on our findings and prior studies on oxidation reactions catalyzed by copper enzymes,^[^ ^22^, ^23^, ^24^, ^25^ ^]^ we propose a reaction pathway for CctR‐mediated hydroxylation (Figure S15). Upon binding of two Cu(II) ions to the enzyme, two electrons are transferred to the copper center, reducing both Cu(II) ions to Cu(I). O_2_ then binds to the reduced copper, forming a cupric–superoxide intermediate. This reactive species abstracts a hydrogen atom from the C(*sp* ^3^)─H bond of the substrate, generating a carbon radical in the substrate. Two potential pathways may follow for the hydroxylation of this radical intermediate. In the first pathway, the hydroxyl group derived from the hydrogen atom abstraction rebounds to the carbon radical, completing the hydroxylation. In the second pathway, the hydroxyl group is released as a water molecule, and the resulting copper–oxyl species reacts with the carbon radical, which leaves the active center after protonation as the hydroxylated product. We emphasize that this proposed mechanism is hypothetical; detailed elucidation of the catalytic steps will require future investigation using structural, biochemical, and spectroscopic approaches.

To investigate substrate binding of CctR, we conducted a total of 26‐µs all‐atom molecular dynamics (MD) simulations with the substrate cyclochlorotine initiated from four distinct poses predicted by Audock Vina.^[^ ^26^ ^]^ We hypothesized that in a productive binding pose, the β‐carbon (C_β_) in the 2‐aminobutyrate residue of cyclochlorotine would be in the vicinity of the copper ions. Therefore, we extracted simulation frames from the combined 26‐µs MD trajectories where the C_β_–Cu distance was less than 7 Å. These frames were subjected to clustering analysis, and the resulting clusters were ranked based on their C_β_–Cu distances. The representative structures from the top three clusters are shown in Figures S16a–c, which all have C_β_–Cu distances below 5 Å. For these three representative poses, substrate‐contacting residues were identified by measuring CctR‐cyclochlorotine distances, where a residue was considered to be in contact with the substrate if any pair of their atoms was within 3.5 Å. Among the identified residues (Figures S16a–c), five that made contact with cyclochlorotine in at least two poses, namely, Trp108, Ser170, His172, Thr173, and Thr203, were subjected to site‐directed mutagenesis. Of these, only the W108A mutant showed a significant loss of catalytic activity, while the other variants retained activity comparable to the wild‐type enzyme (Figure S16d). These results suggest that either the predicted active‐site pocket residues involved in substrate binding are relatively tolerant to mutation, or that further computational and experimental studies—such as X‐ray crystallography—are needed to fully elucidate the critical binding interactions between cyclochlorotine and CctR.

In summary, our results establish the DUF3328 protein CctR as a representative of a new family of copper‐dependent hydroxylases capable of activating inert C(*sp* ^3^)─H centers. We demonstrate that CctR requires both electron input and molecular dioxygen for catalytic activity. The AlphaFold‐predicted structure provides important insights into DUF3328 protein architecture, particularly the dimeric assembly and the spatial arrangement of the characteristic HxxHC(x)_n_HxxHC motif. During the revision of this manuscript, Chiang et al. reported the biochemical characterization of two DUF3328 enzymes: the halogenase ApnU and the cyclase AprY.^[^ ^27^, ^28^ ^]^ These enzymes were expressed as N‐terminally truncated proteins in *E. coli* and purified through a series of carefully optimized steps, including denaturing purification, protein refolding, and re‐dimerization via disulfide bond reformation. Like CctR, both ApnU and AprY were shown to be copper‐ and dioxygen‐dependent and to require dimerization for function. DUF3328‐family proteins, thus far found exclusively in fungi, have previously been investigated using genetic approaches such as gene knockouts and heterologous expression. These studies suggest that members of this family catalyze a range of reactions, including halogenation, oxidation, isomerization, and macrocyclization.^[^ ^5^, ^6^, ^7^, ^8^, ^9^ ^]^ DUF3328 thus represents an underexplored enzyme family harboring rich catalytic potential and largely uncharacterized mechanisms. This remarkable functional diversity not only opens new avenues for enzyme engineering in industrial applications, but also offers a unique system for studying the evolutionary processes underlying the divergence of catalytic activities within a protein family. Notably, our characterization of this previously understudied protein family was significantly accelerated by AlphaFold. We anticipate that this AlphaFold‐guided approach to functional and structural characterization will continue to enable the discovery and mechanistic elucidation of novel enzyme families.

## Author Contributions

W.H. and J.‐K.W. designed the research. W.H. performed most of the experiments. J.K.R. conducted NMR analyses. A.T. assisted with molecular genetics experiments. W.H. and X.Z. conducted the ^18^O_2_ labeling experiments. D.B., S.N., J.G. performed the EPR experiments and data analysis. N.G. and A.I. performed the native protein mass spectrometry. B.L. and Y.W. performed the molecular docking and simulations. W.H. and J.‐K.W. wrote the manuscript with input from all authors.

## Conflict of Interests

J.‐K.W. is a member of the Scientific Advisory Board and a shareholder of DoubleRainbow Biosciences, Galixir, and Inari Agriculture, which develop biotechnologies related to natural products, drug discovery and agriculture. All other authors have no competing interests.

## Supporting information



Supporting Information

## Data Availability

The data that support the findings of this study are available in the Supporting Information of this article.
