# Supplementary material for: Cyclochlorotine Hydroxylase CctR Reveals DUF3328 as a Family of Copper‐Dependent Metalloenzymes
Source: Angew Chem Int Ed Engl. 2025 Aug 22;64(38):e202512449. doi: 10.1002/anie.202512449 (PMC12435403; doi:10.1002/anie.202512449)
Supplement: Supplementary file 1 — Supporting Information [file ANIE-64-e202512449-s001.pdf]

Supporting Information

Cyclochlorotine Hydroxylase CctR Reveals DUF3328 as a Family of Copper-Dependent Metalloenzyme

Wentao Huang[1,2,3,4,5], Jakob K. Reinhardt[1,3,4], Anru Tian[5,6], Xiao Zhang[7], Binghui Li[8], Noah Gould[3,9], Sashirekha Nallapati[3], Alexander R. Ivanov[3,9], Yi Wang[8], Jason J. Guo[3], David E. Budil[3], Jing-Ke Weng[1,3,4,5,10,*]

[1]Institute for Plant-Human Interface, Northeastern University, Boston, MA 02115, USA

[2]Department of Biology, Massachusetts Institute of Technology, Cambridge, MA 02139, USA

[3]Department of Chemistry and Chemical Biology, Northeastern University, Boston, MA 02115, USA

[4]Department of Bioengineering, Northeastern University, Boston, MA 02115, USA

[5]Whitehead Institute for Biomedical Research, Cambridge, MA 02142, USA

[6]Department of Biological Engineering, Massachusetts Institute of Technology, Cambridge, MA 02139, USA

[7]Department of Chemistry, Massachusetts Institute of Technology, Cambridge, MA 02139, USA

[8]Department of Physics, The Chinese University of Hong Kong, Hong Kong, P. R. China

[9]Barnett Institute of Chemical and Biological Analysis, Northeastern University, Boston, MA, 02115, USA

[10]Department of Chemical Engineering, Northeastern University, Boston, MA 02115, USA

*Correspondence author:

Jing-Ke Weng, Email: jingke.weng@northeastern.edu

Table of Contents

Materials and Methods…………………………………………………………………..…...…3

Supplementary Figures……………………………………………………………………...10

Supplementary Tables………………………………………………………………………38

References…………………………………………………………………………………..42

Materials and Methods

Strains and culture conditions

Talaromyces islandicus (formerly *Penicillium islandicum*) was obtained from ATCC (ATCC® 10127™). The fungus was cultured under various conditions: on potato dextrose agar (PDA, Sigma 70139) plates at 30 °C for solid culture; in potato dextrose broth (PDB, Sigma P6685) at 30 °C with 200 rpm shaking for rapid growth; and in static Czapek yeast extract medium (prepared according to standard recipe) at 30 °C for production of cyclochlorotine derivatives.

Liquid chromatography–mass spectrometry (LC-MS) analysis of cyclochlorotine derivatives

UHPLC-Mass spectrometry system (Vanquish Flex Binary UHPLC, Orbitrap Exploris 120, Thermo Fisher) was used to detect the cyclochlorotine derivatives. The column used in UHPLC is the Kinetex 2.6um C18 100A 150*3 mm column (Phenomenex, part no. 00F-4462-Y0). UHPLC method: Buffer A is water + 0.1% formic acid, Buffer B is acetonitrile + 0.1% formic acid. Flow rate is 0.5 mL/min. Column temperature is 35 ℃. Gradient is 0-2 min: 5% B, 2-15 min: 5-50% B, 15-20 min: 95% B, 20-25 min, 5% B. Mass spectrometry method: MS1 full scan 100-1000 m/z, positive mode, resolution at 60,000. LC/MS data was analyzed by Freestyle software (Thermo Fisher) and mzmine[1]. $[M+H]^+$ adduct was extracted for each derivative within 5 ppm accuracy window.

Purification of cyclochlorotine and hydroxycyclocholorotine

T. islandicus was cultured in Czapek Yeast Extract medium without agar at 30 ℃ without shaking for three weeks. The supernatant was repeatedly sampled to monitor the production of cyclochlorotine and its derivatives (data not shown). At around 3 weeks, production will reach saturation. The culture was then filtered, and the supernatant used for compound purification. Granulated activated charcoal (Sigma, C3014) was added (5 g per 100 mL) and the mixture was shaken at 150 rpm, 30 °C, for 3 h. Separating the charcoal from the medium was achieved by centrifugation at 4300 g for 8 min. The charcoal was washed twice with water (2x 100 mL per 100 mL medium) to remove residual medium. The activated charcoal was extracted three times (standing with the solvent for 2 h, 15 h, and again 2 h for the final extraction) with acetone (per extraction 50 mL acetone/100 mL medium) and the acetone extracts were pooled. The combined acetone extracts were dried in vacuo and dissolved in methanol for chromatography on LH-20 resin in MeOH (glass column, l=35 cm, d=3 cm). Fractions were analyzed on mass spectrometry and fractions containing the compound of interest were collected and dried. The dried fractions were redissolved in methanol and injected into preparative HPLC (Shimadzu) for further purification. For the preparative HPLC, buffer A is water + 0.1% formic acid, and buffer B is acetonitrile + 0.1% formic acid. Column is Kinetex 5 µm C18 100 Å, LC Column 250 x 10.0 mm (cat no. 00G-4601-N0), and flow rate is set to be 3 mL/min with absorption monitored at 222 nm. Separation of the pooled fractions containing cyclochlorotine using an isocratic flow of 22% B yielded pure cyclochlorotine. Separation of the pooled fractions containing hydroxycyclochlorotine using an isocratic flow of 18% B yielded pure hydroxycyclochlorotine. Structure of cyclochlorotine and hydroxycyclochlorotine were confirmed using nuclear magnetic resonance (NMR).

NMR of cyclochlorotine and hydroxycyclocholorotine

Chemical structures of cyclochlorotine and hydroxycyclochlorotine were identified using NMR. The corresponding data are listed in Figure S17-S27 and Table S2. NMR spectra were recorded on a 700 MHz Bruker AVANCE-Neo NMR spectrometer in DMSO-d_6 (Cambridge Isotope Laboratories Inc.) at 298 K using a cryoprobe. Next to 1H-NMR spectra, DEPT-Q-13C, COSY, HSQC, and HMBC spectra were acquired. For hydroxycyclochlorotine, also a ROESY spectrum was recorded. NMR data was analyzed using TopSpin 4.4.1. The residual solvent signals of DMSO (δ_H 2.50 ppm, δ_C 39.52 ppm) were used for referencing the 1D spectra. For cyclochlorotine, two sets of NMR signals were observed corresponding to two different stable conformers in DMSO. Using the integrals of the Abu^2-γ methyl group (3 H, δ_H 0.84 ppm, t (7.4 Hz) and 1.59 H, 0.91 ppm, t (7.4 Hz)), the ratio between the conformers was determined as approximately 2:1. While the chemical shifts for both conformers correspond to the data reported by Mizutani et al.[2], the major conformer in this study corresponds to the conformer reported as minor and vice versa. While this confirms the structure of cyclochlorotine, it implies a certain variability in conformer populations even in the same solvent at the same temperature. Differences in chemical shifts were observed for exchangeable NH protons. For example, Phe^4-NH was found at δ_H 8.53 and 7.85 ppm here for the major and the minor conformer, respectively, but corresponding signals were reported at δ_H 8.19 and 7.76 ppm. This, together with the presence of residual formic acid in the sample could hint at a pH dependent distribution of conformers. Similarly, for hydroxycyclochlorotine, two sets of ^{1}H and ^{13}C NMR signals were observed with a ratio of 3:1 based on the integrals of the *allo*Thr^2-γ methyl group (3 H, δ_H 1.21 ppm, d (6.1 Hz) and 1.59 H, 1.00 ppm, d (6.6 Hz)). Chemical shifts observed for the major conformer correspond well with reported values for hydroxycyclochlorotine and thus confirm the identity as hydroxycyclochlorotine[2]. While no other conformers were previously reported, the generally lower abundance of this conformer together with the apparent variability in conformers under these conditions would explain this difference. ROESY analysis of the major hydroxycyclochlorotine conformer showed ROE contacts between *allo*Thr^2-β (δ_H 4.17 ppm) and both Ser^3-NH (δ_H 8.89 ppm) and *allo*Thr^2-NH (δ_H 8.46 ppm), as well as between *allo*Thr^2-γ (δ_H 1.21 ppm) and alloThr^2-α (δ_H 4.26 ppm), implying an impeded rotability of the *allo*Thr sidechain. The same pattern was described for hydroxycyclochlorotine by Mizutani et al[2].

RNA-seq of *T. islandicus*

Total RNA was extracted from *T. islandicus* using TRIzol™ Plus RNA Purification Kit (Thermo Fisher, #12183555) according to the manufacturer's protocol. Fungal cells were lysed using TissueLyser II (Qiagen) with metal beads. Library construction and Illumina paired-end sequencing (150 bp) were performed at the Whitehead Genome Core. Sequencing reads were aligned to the *T. islandicus* genome (GCA_000985935.1) using STAR, and alignments were visualized using IGV genome browser[3]. Read coverage across the cyclochlorotine biosynthetic gene cluster was examined in detail. The RNA-seq data was uploaded to NCBI with BioProject ID PRJNA1289731.

Genomic knockout of DUF3328 genes in *T. islandicus* and genetic complementation

The knockout of DUF3328 genes was carried out using CRISPR/Cas9 technique. The experimental design was followed by procedures described by Salazar-Cerezo *et al.*[4]. The involved plasmids were acquired from Addgene (pFC332, Addgene cat# 87845; pFC334, Addgene cat# 87846). The sgRNA sequences targeting *cctP2*, *cctO*, *cctR* were designed using CRISPOR[5]. Three sgRNAs were designed for each gene. The sgRNA sequences used in the experiment are

listed in Table S3. After the CRISPR/Cas9 plasmids were made, they were transformed into *T. islandicus* using the protoplast transformation method described by Schafhauser et al.[6]. Transformants were selected on potato dextrose agar plates containing 500 μg/mL hygromycin. Genomic DNA was extracted from individual colonies and PCR-amplified using gene-specific primers. PCR products were analyzed by Sanger sequencing to confirm successful gene deletion. For complementation, *cctR* was expressed in *T. islandicus* using the CRISPR/Cas9 plasmid backbone with the Cas9 sequence replaced by *cctR*. Transformation was performed using the same procedure as described above.

Computational modeling and analysis of CctR

Phobius analysis was performed on their online server (https://phobius.sbc.su.se/). The initial AlphaFold protein structure model of CctR was generated using colabfold (https://github.com/sokrypton/ColabFold) based on AlphaFold 2. After AlphaFold 3 was released, we updated the model with AlphaFold 3 (https://alphafoldserver.com/). We set the parameters to generate a homodimer structure for CctR. For homodimer structure with Cu^{2+} bound, four copies of Cu^{2+} ion were specified in the program parameters. Among the models generated by AlphaFold 3, the one with the highest score was chosen. The structure files were included as supplementary files.

Recombinant expression of CctR in Sf9 insect cells

The amino acid sequence of DUF3328 protein is listed in Table S4. Sf9 cells in Sf-900™ III SFM was purchased from Thermo Fisher (cat# 12659017). The Sf9 cells were cultured in SFM III medium (Thermo Fisher, cat# 12658027) without antibiotics at 26 °C 130 rpm. Bac-to-Bac™ Baculovirus Expression System (Thermo Fisher, cat# 10359016) was used for making the recombinant DUF3328 protein in Sf9 cells by following their user manual. In summary, the pFastbac plasmid was inserted with c-terminal twin-strep tagged DUF3328 protein sequence. The completed plasmid was transformed into DH10Bac *E. coli* and grown on LB plates containing e, 100 μg/mL X-gal and 40 μg/mL IPTG. After 24 hours, the white colony was picked for BacMid DNA preparation. To make the P1 virus in Sf9 cells, 2 ml of Sf9 cells at 1 million/ml density were plated on a well in a 6-well plate. After letting the cells sit for 15 min, the supernatant medium was removed and replaced with 800 μL fresh SFM III medium. 2 ug of the purified BacMid was mixed with 194 μL SFM III medium and 6 μL FuGENE® HD Transfection Reagent (Promega, cat# E2311). The whole mixture was incubated at room temperature for 15 min and then added to the well. The plate was sealed with parafilm and incubated overnight at 26 °C. Another 1 mL fresh SFM III medium was added to the well next day. Incubate it for another 72 hours, then collect the medium and spin it down at 3,000 g for 5 min to save the supernatant containing the P1 virus. The P1 virus supernatant was then added to a flask of 50 mL Sf9 cells in SFM III medium at density of 1 million/mL to make P2 virus. They were grown at 26 °C 130 rpm for 72 hours. The culture was centrifuged, and the supernatant contained the P2 virus. The supernatant was evenly distributed to 4 flasks each with 1 L Sf9 cells in SFM III medium at density of 1 million/mL. The 4 L culture was grown at 26 °C 130 rpm for 72 hours for final protein expression. After that, the culture was centrifuged down at 3,000 g and the pellet was used for protein purification.

Protein purification from Sf9 insect cells

To solubilize the membrane protein, we have attempted two different purification methods. In the first method, we obtained the membrane fraction through ultra-centrifugation and then solubilize

the membrane fraction with detergent. 4 L of Sf9 cell pellet was dissolved in 150 mL lysis buffer (50 mM Tris/HCl pH 8.0, 0.2 M NaCl, 10% Glycerol, 1X Halt Protease Inhibitor Cocktail (Thermo Fisher, cat# 78438), 2 mM EDTA). The solution was sonicated for ~3-4 min until the solution became homogenized and cells were completely lysed. The solution was then centrifuged down at 10,000 g for 10 min. The supernatant was kept and transferred to an ultra-centrifugation tube. The supernatant was then ultracentrifuged for 1 hour at 120,000 g. The pellet which contains microsome fractions were saved and dissolved in 20 mL resuspension buffer (50 mM Tris/HCl pH 8.0, 0.2 M NaCl, 10% Glycerol, 1% DDM, 1 mM EDTA). Needle and syringe were used to pipette the solution up and down to homogenize the solution. After the solution was completely homogenized, the solution was then sent to centrifuge for another 1 hour at 48,400 g. The supernatant was then applied to a gravity column with 0.5 mL Strep-Tactin® Sepharose® resin (iba, cat# 2-1201-002). In the second approach, we added 1% DDM directly to the lysis buffer to solubilize the membrane protein during lysis step. The lysate was sonicated for ~3-4 min until the solution became homogenized and cells were completely lysed. Then the solution was then sent to centrifuge for another 1 hour at 48,400 g. The supernatant was then applied to a gravity column with 0.5 mL Strep-Tactin® Sepharose® resin (iba, cat# 2-1201-002). Since traditional reducing agents like DTT cannot be used in the for our lysis step, we observed that adding 50mM ascorbate during the lysis step can help increase the stability of the protein. The gravity column was then washed with 3 mL wash buffer (100 mM Tris/HCl pH 8.0, 150 mM NaCl, 1 mM EDTA, 10% Glycerol, 0.1% DDM). The protein was eluted from the column with 3 mL elution buffer (100 mM Tris/HCl pH 8.0, 150 mM NaCl, 1 mM EDTA, 2.5 mM desthiobiotin, 10% Glycerol, 0.1% DDM). The eluted protein was buffer exchanged to low-salt anion-exchange column buffer A (10mM Tris/HCl pH 8.0, 10 mM NaCl, 10% Glycerol, 0.1% DDM) before loading it to anion exchange column (Resource Q, 1ml, Cytiva) on AKTA Pure 25 protein purification system (GE). A gradient of 0-100% buffer B (10mM Tris/HCl pH 8.0, 1M NaCl, 10% Glycerol, 0.1% DDM) was applied for a total of 40 column volume. The fractions containing CctR protein were concentrated to 200 µL and were applied to gel-filtration column (Superdex 200 increase 10/300 gl column, Cytiva) on AKTA Pure 25 system for the final purification (FPLC buffer: 50 mM Tris/HCl pH 8.0, 150 mM NaCl, 10% Glycerol, 0.1% DDM). The fractions containing the protein was then collected and concentrated for storage at -80 °C.

Blue-Native PAGE gel

The reagents for Blue-Native PAGE gel was purchased from Thermo Fisher, which includes the NativePAGE™ Bis-Tris Mini Protein Gels, 4 to 16%, 1.0 mm (Cat# BN1002BOX), NativeMark™ Unstained Protein Standard (Cat# LC0725), NativePAGE™ Running Buffer (20X) (Cat# BN2001), NativePAGE™ Cathode Buffer Additive (20X) (Cat# BN2002), NativePAGE™ 5% G-250 Sample Additive (Cat# BN2004), NativePAGE™ Sample Buffer (4X) (Cat# BN2003). The gel was run according to the protocol provided by Thermo Fisher.

Native protein mass spectrometry

For native protein mass spectrometry, the detergent used for CctR protein purification was tetraethylene monooctyle ether (C8E4) instead of DDM. Before loading the sample to mass spectrometer, the protein was buffer exchanged into 200 mM ammonium acetate with 0.5% C8E4 using a Bio-Rad Micro Bio-Spin P-6 Gel Column, as directed by the manufacturer, with a final concentration of 0.06 mg/mL. 10 microliters of sample was then transferred to a gold-coated glass nanoflow capillary (type Medium NanoES spray capillaries for the Micromass Q-Tof, Odense,

Denmark) and sample was introduced to the mass spectrometer using a Nanospray Flex Ion Source (Thermo Scientific) in offline configuration. CctR protein samples were analyzed using a Q-Exactive™ UHMR (Ultrahigh Mass Range) Hybrid Quadrupole Orbitrap™ Mass Spectrometer (Thermo Scientific). For native mass spectrometry analysis, the source spray voltage was set to +1.6 kV with a capillary temp of 225 °C. Ion transfer target *m/z* was set to "low *m/z*" and the detector optimization was set to "high *m/z*". The in-source trapping voltage was set at -100 to -300 V, depending on the analysis and observed protein solvation, and the trapping gas pressure was set to 3. Data was acquired at a resolution setting of 6250, at *m/z* 400, with a *m/z* range of 2000-10000. Data analysis and mass spectrum deconvolution were performed using UniDec[7]. For CctR, deconvolution settings included a *m/z* range of 3500 to 7000, a charge range of 10 to 30, an intensity threshold of 0.05, and a mass range of 30000-100000 Da, sampled every 1 Da. The peak fwhm was set to 0.85 Th and the charge smooth width and point smooth width were set to 1.

In vitro enzyme assay of CctR

In the metal screening assay, each reaction was performed in 20 µL final volume containing 5 µM protein in reaction buffer (50 mM Tris/HCl pH 8.0, 100 mM NaCl, 0.05% DDM), with substrates at final concentration of 0.5 µg/µL and each metal at 1mM concentration. Reactions proceeded overnight and were quenched with 100 µL MeOH. The solutions were centrifuged at 21,000 g for 5 min to remove precipitates, and the supernatants were analyzed by LC/MS. For reducing agent assay, NADPH, NADH, and ascorbic acid were added to 10 mM final concentration with 1mM Cu^{2+}, 5 µM enzyme and 0.5 µg/µL substrate. For BCA experiments, bicinchoninic acid disodium salt was dissolved in reaction buffer and added to a final concentration indicated in each reaction. For $^{18}O_2$ experiments, reactions were set up in a Schlenk flask and immediately frozen in liquid nitrogen. The flask underwent two freeze-pump-thaw cycles under high vacuum to remove air, followed by introduction of $^{18}O_2$ (Sigma, 490474). For all CctR mutants' assay, 5 µM of protein, 10 µM Cu^{2+}, 10mM Ascorbate and 0.05 µg/µL substrate are used in each reaction. Because there are several types of assays in the manuscript and they are conducted and analyzed by LC/MS at different times, the retention time of substrate and product are slightly shifted in different LC/MS batches. Thus, compound standard was used in each LC/MS run to validate the peak and retention time.

Molecular dynamics of CctR and substrate binding

All MD simulations were performed using GROMACS[8] 2022.6 with the CHARMM36m force field[9] for the CctR protein and the CGENFF force field[10] for the cyclochlorotine substrate. The initial structure of CctR was predicted by AlphaFold3. To enhance simulation efficiency, a truncated version comprising residues 70 to 261 was used. Cu ions were modeled as monovalent.

Cyclochlorotine conformations were generated through simulated annealing. To model this system, a single cyclochlorotine molecule was solvated in a dodecahedron box with a 1.2 nm margin of TIP3P water molecules and neutralized with 0.15 M NaCl. The system was subjected to energy minimization, followed by 200 ps of NVT equilibration. Simulated annealing was then performed starting from 300 K for 20 ns, followed by heating to 365 K at a rate of 6.5 K/ns. After an additional 20 ns at high temperature, the system was cooled back to 300 K at the same rate. Each complete heating–cooling cycle lasted 60 ns and the process was repeated periodically. The system temperature was controlled by the velocity-rescaling thermostat[11]. A total of 900 ns of simulated annealing was performed. Cluster analysis was then applied to the trajectories at 300 K, and the representative conformations from the top four clusters were selected for docking to CctR

using AutoDock Vina 1.1.2[12], resulting in four initial models of the CctR–cyclochlorotine complex.

Each initial model of the complex was solvated in a dodecahedron box with a 1.2 nm margin of TIP3P water molecules and neutralized with 0.15 M NaCl. The four systems were then subjected to energy minimization, followed by 200 ps of NVT equilibration and 200 ps of NPT equilibration. Subsequently, four replicas of production MD simulations ranging from 1 to 2 μs were carried out for each system in the NPT ensemble, resulting in a total simulation time of 26 μs. During the simulations, the system temperature was maintained at 300 K using the velocity-rescaling thermostat, and the pressure was maintained at 1 bar using the C-rescale barostat[13]. Positional restraints were applied to the Cu ions, the side chains of His146, His149, His176, His179, Cys150, and Cys180, as well as to residues forming β-sheet or α-helix secondary structures. Tyr230, Thr231, Gly235, and Phe236 were exceptions because of the short length of the sheet and their proximity to the binding site.

In all simulation, van der waals interactions were smoothly switched off between 1.0 and 1.2 nm. Electrostatic interactions were treated using the particle mesh Ewald (PME) method[14] with a cutoff of 1.2 nm. All bonds involving hydrogen atoms were constrained using the LINCS algorithm[15]. Visualizations were prepared using VMD[16].

Stoichiometry of Cu to protein quantification using ICP-MS

For the samples used for Cu quantification, HEPES was used to replace Tris/HCl in the sample buffer, since Tris has a slight affinity to metal ions. The CctR protein concentration was quantified using the CBQCA Plus Protein Quantitation Kit (Thermo Fisher, cat# A66522), which is a protein quantification kit for low concentration membrane protein and is also compatible with detergent. The concentration of CctR was then normalized to 2 μM (monomeric count), and incubate with 8 μM (4 equivalence, batch 1) and 10 μM (5 equivalence, batch 2) of Cu^{2+} for 2 hours at 4 °C to allow for sufficient binding to protein. After that, the solution was transferred to Pur-A-Lyzer™ Midi Dialysis Kit (Sigma, #PURD60030) for dialysis for 3 days, with the dialysis buffer refreshed every day. After dialysis, the solution was transferred out and the new expanded volume was measured. The sample was then sent to Dartmouth Trace Element Analysis Core Facility for ICP-MS analysis to quantify the copper concentration. The molar ratio of Cu to protein was then calculated.

HYSCORE/EPR experiments and data analysis

5 μM CctR protein was incubated with 25 μM Cu^{2+} for 4 hours and then buffer exchanged several times to remove excess amount of unbound Cu^{2+} using centrifugal filter units (MilliporeSigma, Amicon Ultra-15 Centrifugal Filter Units, 30 KDa, cat# UFC9030). After removing the excess amount of unbound Cu^{2+}, the sample was further concentrated down to 100 µl at 100 μM. The sample was then transferred to 3 mm thin wall precision suprasil EPR Sample Tube (Wilmad-LabGlass, cat# 725-PQ-159M), and analyzed by continuous wave (CW) EPR spectroscopy on a local Bruker EMX spectrometer followed by hyperfine sublevel correlation (HYSCORE) spectroscopy[17] on an ELEXSYS E580 EPR instrument on the Bruker Biospin campus (Billerica, MA).

CW-EPR spectra were obtained using a TE102 cavity with microwave frequency and power of 9.5141 GHz, and 6.39 mW respectively and 1.5 mT field modulation at 100 kHz. Nine 1024 point scans were taken with a field sweep time of 83 s and a time constant of 164 ms. The sample was maintained at 139 K using cold nitrogen gas flow.

HYSCORE data were acquired at with a static field set at 307.0 mT, and microwave irradiation at a microwave frequency of 9.7210 GHz. Pulse lengths were 16 and 32 ns for $\pi/2$ and π pulses, respectively and τ was set at 152 ns. 256 points were collected along each time dimension with time steps $t_1 = t_2 = 16$ ns, and a 4-step phase cycle was used to eliminate undesired signals. Data were processed using the Matlab plugin HYSCOREAN[18], employing zero-filling, 1st order polynomial background correction, and Chebyshev apodization; noise interference was adjusted by changing the minimum contour level. Sample temperature was maintained at 20K using a helium flow cryostat.

Supplementary Figures

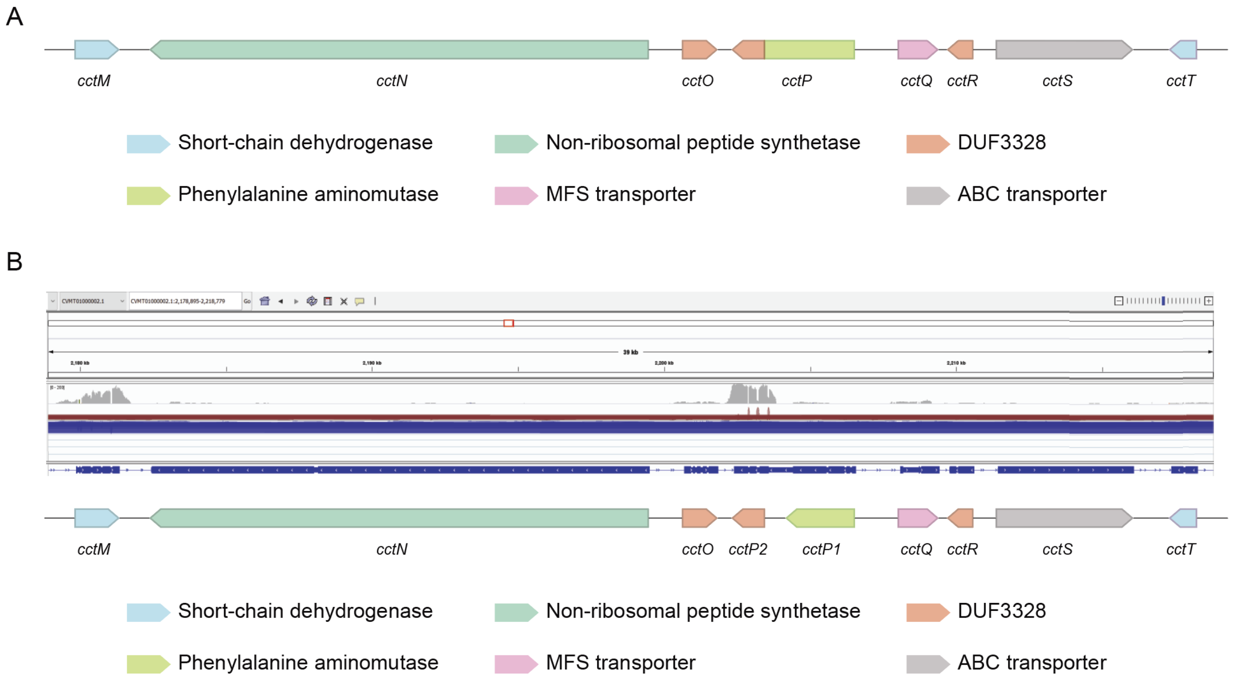

Figure S1. Revision of the cyclochlorotine biosynthetic gene cluster based on RNA-seq analysis. (**A**) Configuration of the original cyclochlorotine biosynthetic gene cluster identified by Schafhauser et al. Please note that they originally named the phenylalanine aminomutase and a DUF3328 protein as a fusion gene *cctP*. (**B**) Top: Our RNA-seq results visualized by IGV genome browser. The image shows the read coverage of the *cctP* genomic region in *T. islandicus*. Read distribution reveals *cctP* contains two independently expressed genes: *cctP1* (phenylalanine aminomutase) and *cctP2* (DUF3328). Bottom: The revised biosynthetic gene cluster of cyclochlorotine.

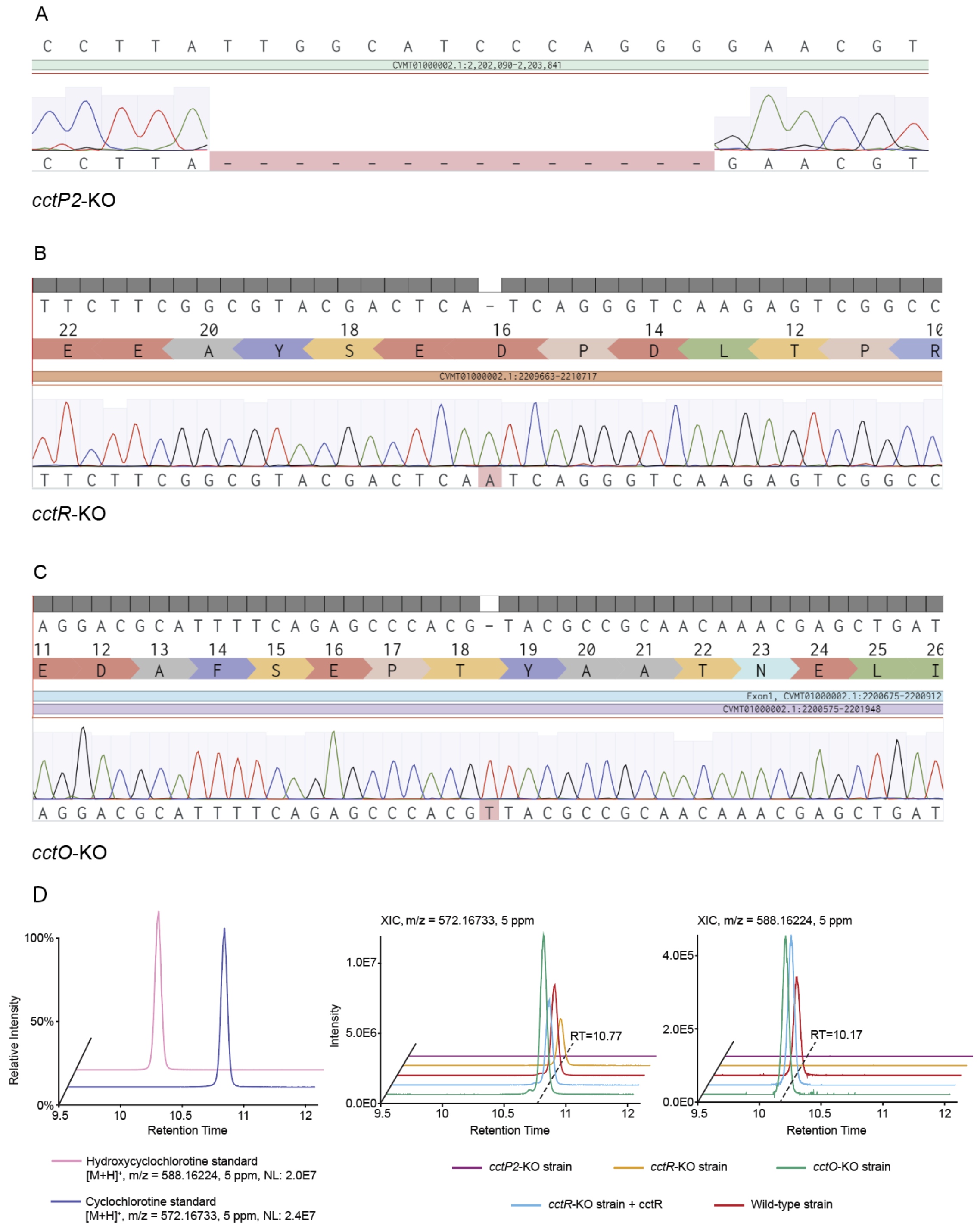

Figure S2. Genetic disruption and functional complementation of DUF3328 genes in cyclochlorotine biosynthesis. (**A-C**) Sanger sequencing confirms CRISPR/Cas9-mediated disruption of DUF3328 genes. (**D**) Extracted ion chromatograms showing the abundance of cyclochlorotine and hydroxycyclochlorotine in the wild-type strain, three *DUF3328*-KO strains, and the *cctR*-KO strain transformed with a plasmid containing a functional *cctR* gene expression cassette.

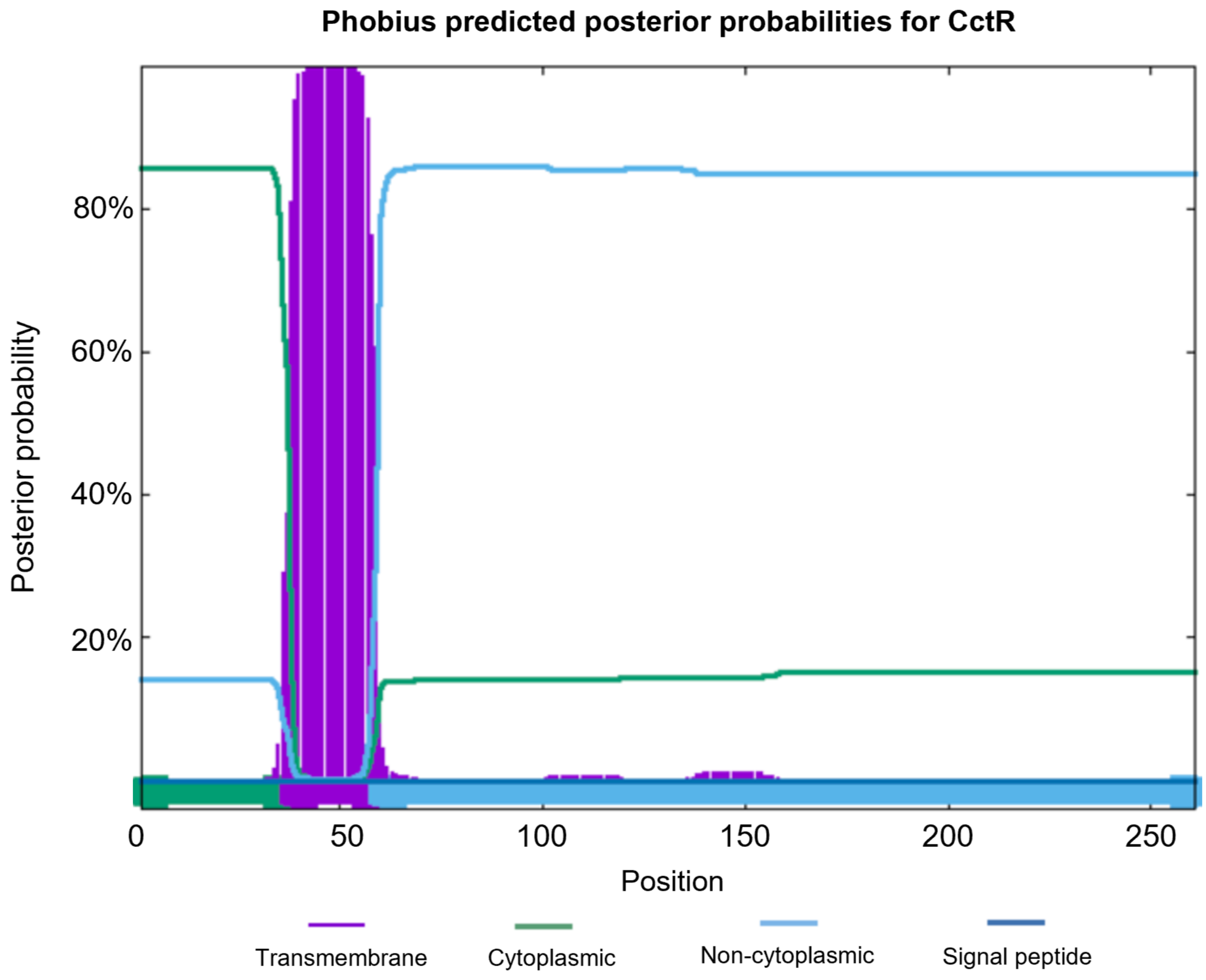

Start	End	Prediction
1	37	Cytoplasmic
38	59	Transmembrane
60	261	Non-cytoplasmic

Figure S3. Phobius[19] prediction of transmembrane topology and signal peptides in the CctR protein sequence. There is a single transmembrane domain in the N-terminus of CctR. The majority of CctR protein is in the C-terminal and predicted to be non-cytoplasmic. No signal peptide was found in the protein sequence.

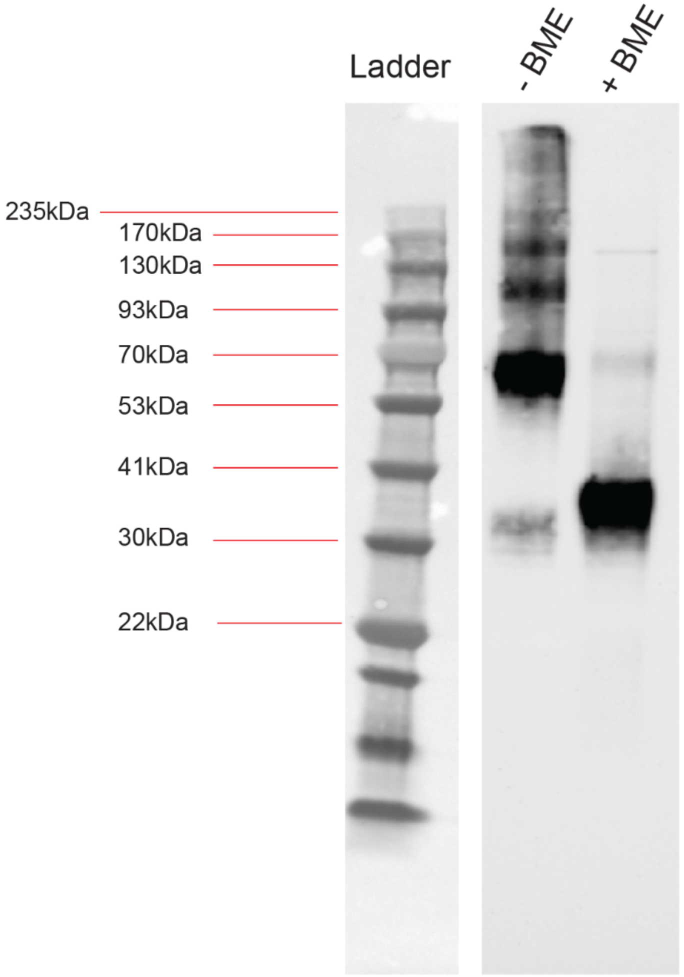

Figure S4. Western blot analysis of C-terminally Twin-Strep-tagged CctR expressed in Sf9 cells. The samples are direct whole cell lysate from strains expressing recombinant CctR protein. The predicted molecular mass of the recombinant protein including tag and linker is 32.87 kDa. Protein was detected using streptavidin-HRP and chemiluminescence. Lanes from left: molecular weight markers, samples without β-mercaptoethanol (BME), samples with BME. CctR predominantly appears at the predicted homodimer mass without BME, but appears at monomer size after being treated with BME. Higher molecular weight bands likely represent protein aggregates due to the disordered region and transmembrane domain. Band smearing is possibly due to post-translational modifications on protein when expressed in the insect cells. Ladder has no chemiluminescence and thus was imaged in epi-white mode (left image), and the samples were imaged in chemiluminescent mode (right image).

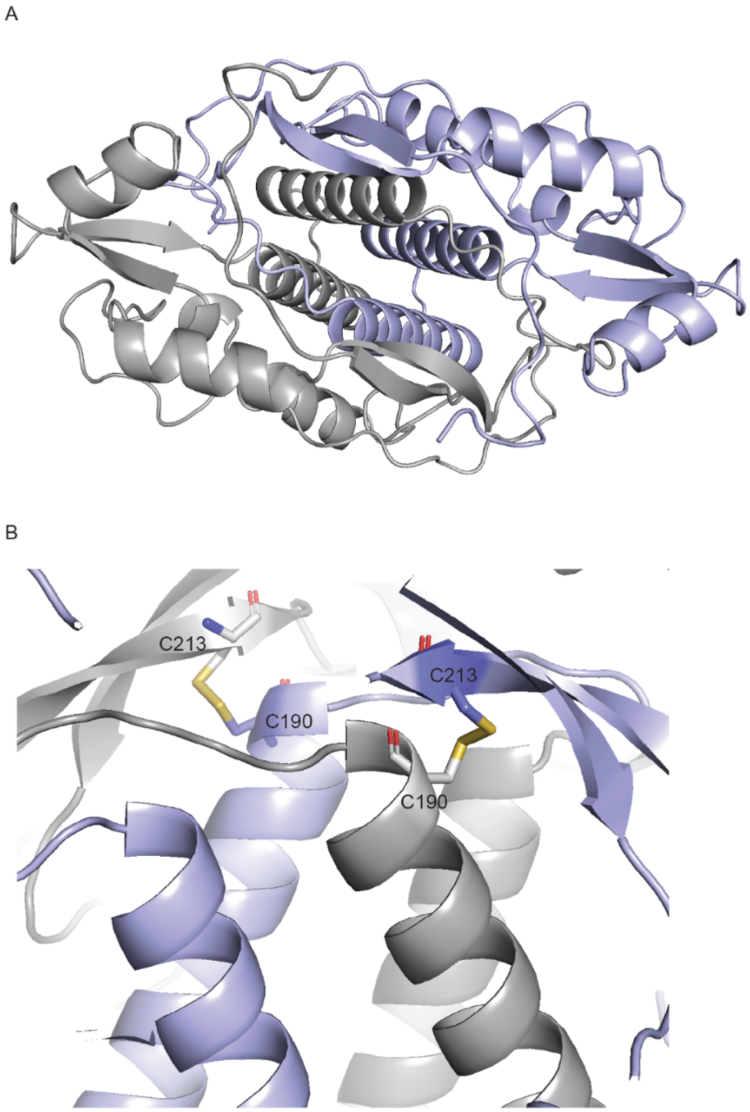

Figure S5. Close-up views of the C-terminal region in the AlphaFold-predicted homodimer structure. (**A**) Four-helix coiled coil at the dimerization interface (N-terminal region omitted for clarity). (**B**) Inter-unit disulfide bonds at the dimerization interface. The two monomers are colored light blue and grey.

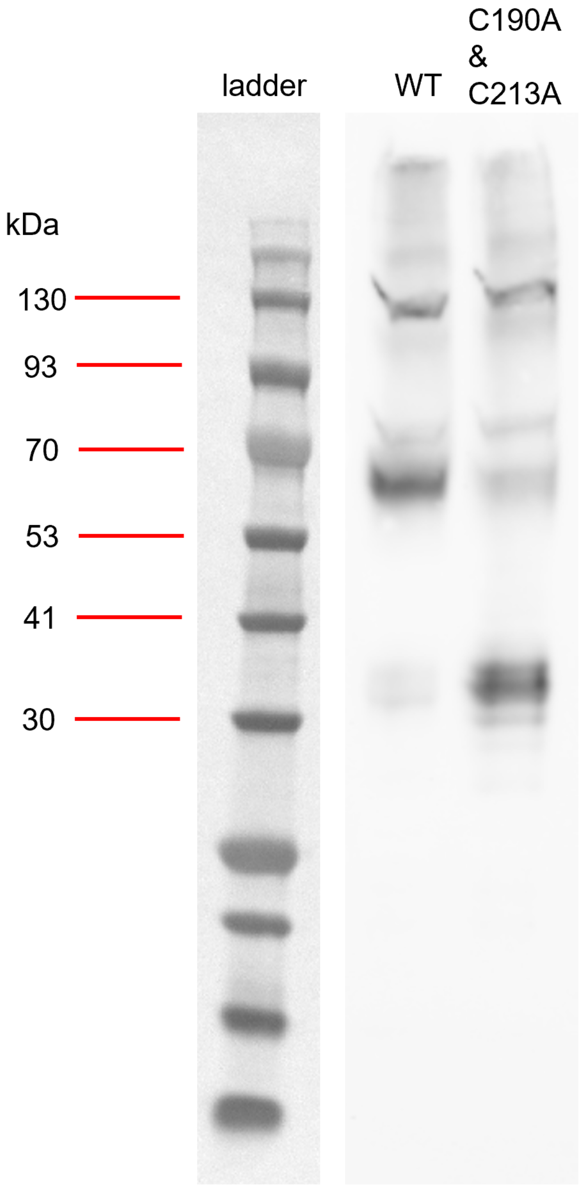

Figure S6. Western blot analysis of C-terminally Twin-Strep-tagged CctR expressed in Sf9 cells. The samples are direct whole cell lysate from strains expressing wild-type CctR protein and C190A & C213A double mutant CctR protein. The dimer band was mostly lost in the double mutant cell line, indicating CctR dimerization is mediated by the disulfide bond formed between C190 and C213. Ladder has no chemiluminescence and thus was imaged in epi-white mode (left image), and the samples were imaged in chemiluminescent mode (right image).

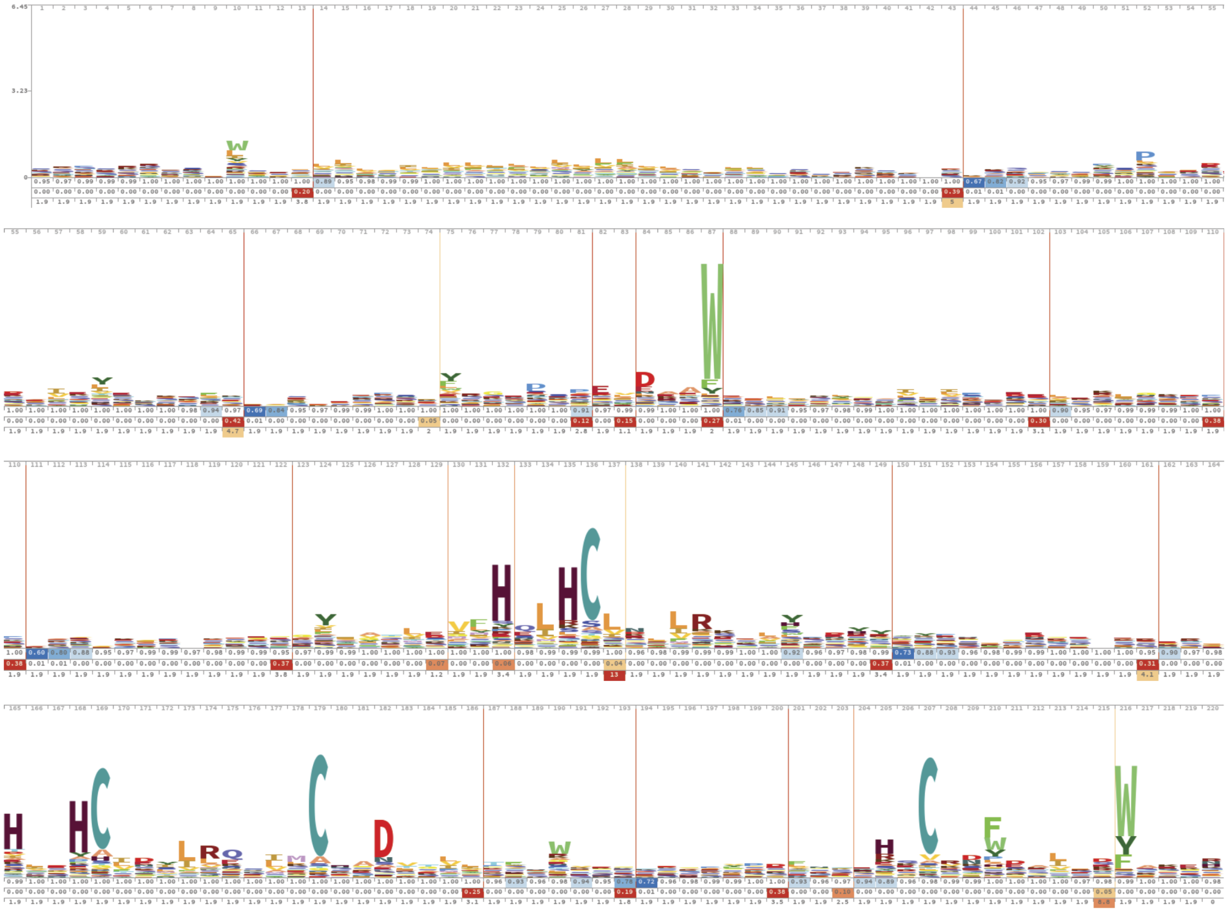

Figure S7. Amino acid sequence logo plot of DUF3328 family. The plot was generated by Skylign[20] using the downloaded HMM[21] profile of DUF3328 family from InterPro (previously Pfam[22] PF11807).

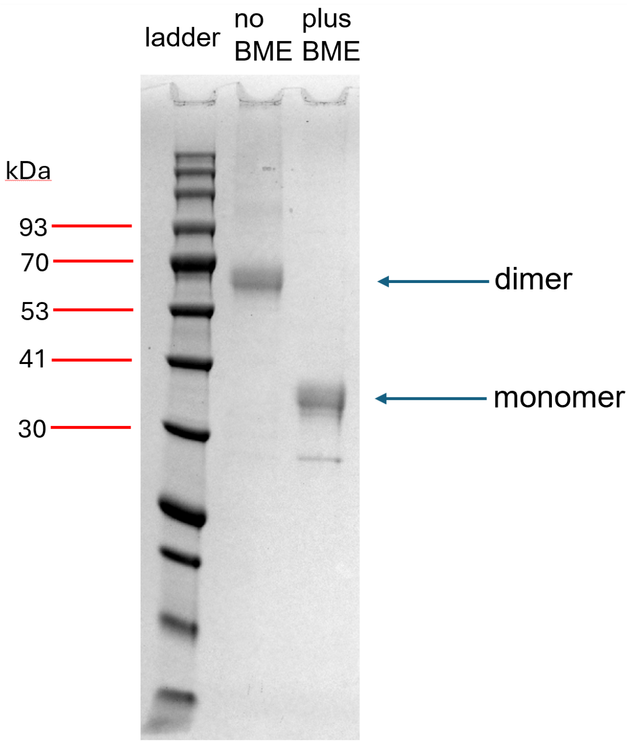

Figure S8. Coomassie-stained SDS-PAGE analysis of purified C-terminally Twin-Strep-tagged CctR. The predicted molecular mass of the recombinant protein including tag and linker is 32.87 kDa. Lanes from left: molecular weight markers, purified protein without BME, purified protein with BME. The smear of the protein band indicates there are some post-translational modifications on CctR protein, which is common for membrane protein expressed in insect cells.

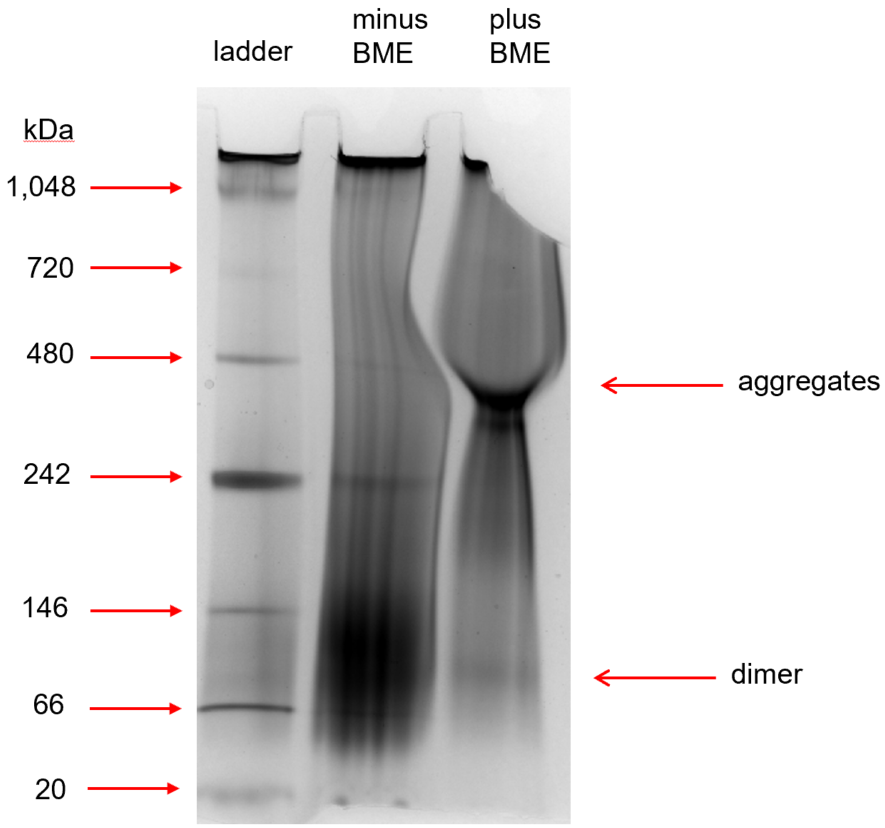

Figure S9. Native CctR protein analyzed by Blue-Native PAGE. In the absence of BME, CctR migrates predominantly in the dimer-size region. Upon addition of BME, the protein does not appear in the monomer region but instead forms high-molecular-weight aggregates, indicating that the monomeric form is unstable.

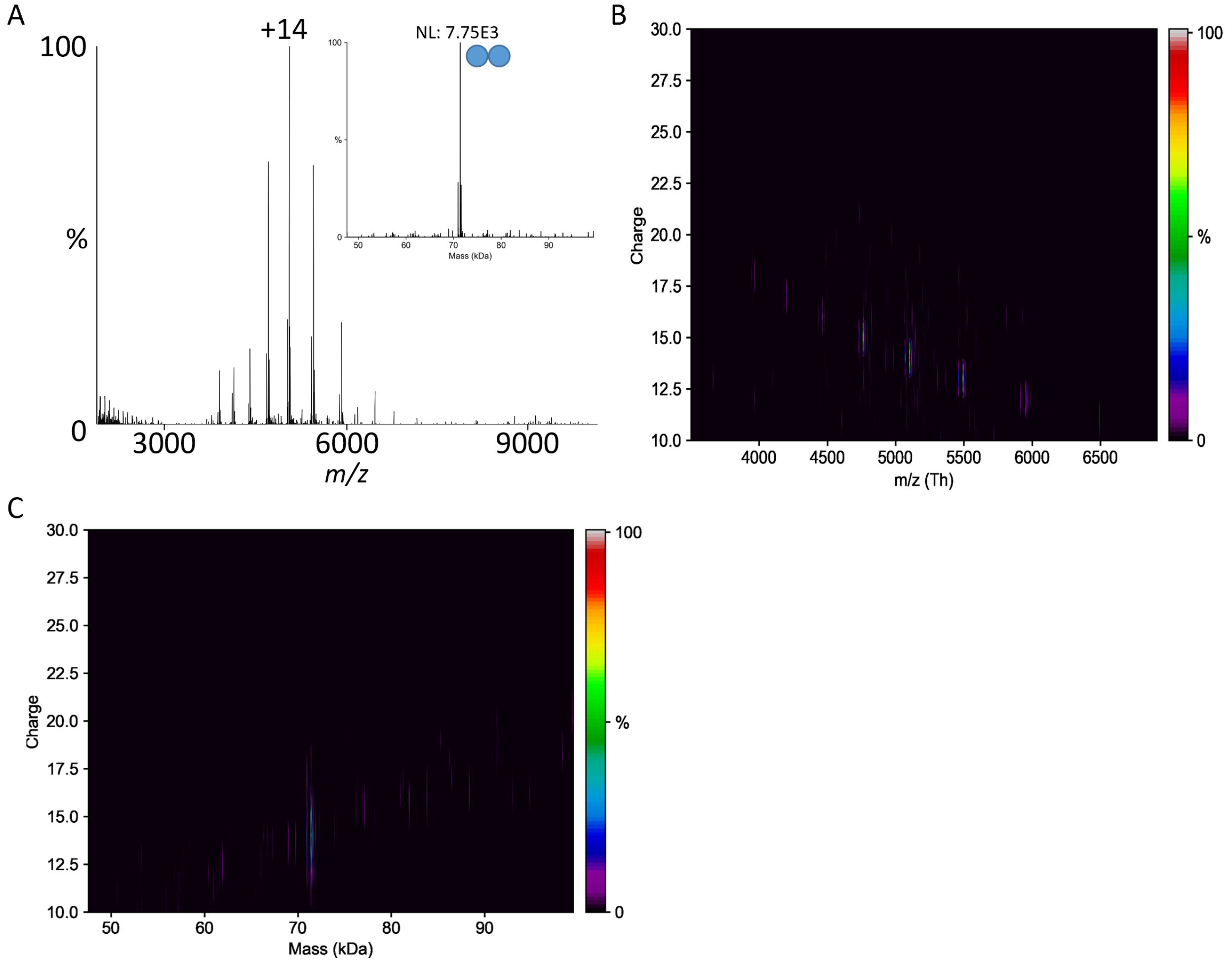

Figure S10. Native mass spectrometry analysis of CctR protein collected at an in-source trapping voltage of -300 V. (**A**) mass spectrum of CctR under native conditions with the charge state of the major distribution annotated, and the inset shows the corresponding deconvolved spectrum. The most abundant mass for the protein complex was 71,416 Da. In addition, there are lower abundant species at 70,965 Da, 71,454 Da, 71,482 Da, and 71,607 Da. The charge state distribution observed for the protein is centered at +14, with a range of +11 to +18. The theorical size of strep-tagged CctR dimer is 65,758 Da. The difference of the protein mass is likely due to the post-translational modifications of the protein when expressed in insect cells. The deconvolved masses are consistent with the gel band ranges observed for the CctR dimer complex, and no monomer or higher-order oligomers were detected. (**B**) UniDec heat map of the observed charge vs *m/z,* with the color corresponding to relative intensity, showing a charge state range of +11 to +18. (**C**) UniDec heat map of the observed charge vs the deconvolved mass.

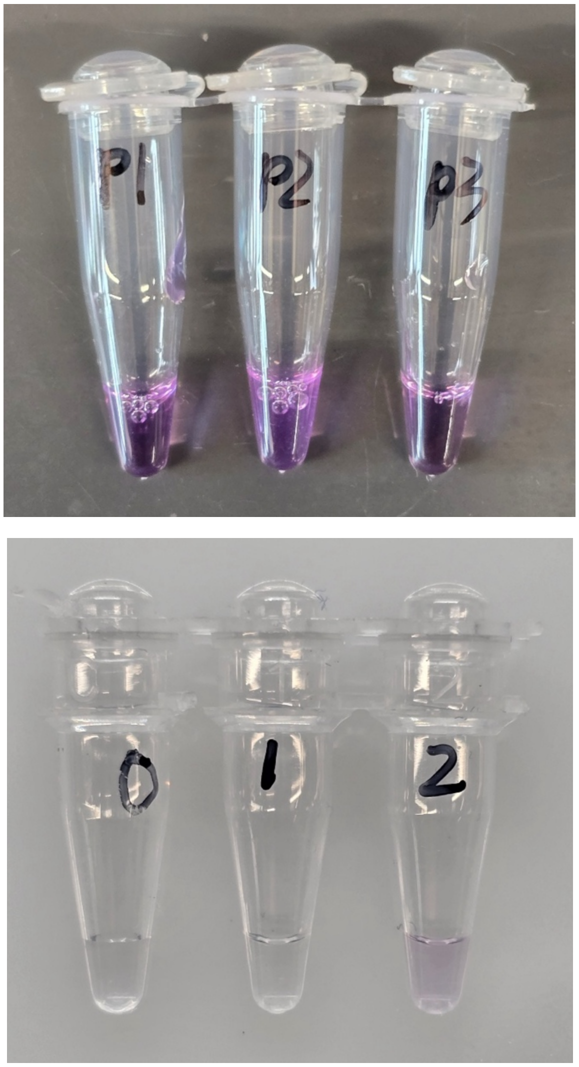

Figure S11. Cu(I) is generated when Cu(II) is incubated with protein. The appearance of a purple color indicates Cu(I) binding to BCA. Top: Three unrelated proteins (2 mg/mL) were incubated with 1 mM Cu(II) and 1 mM BCA. Bottom: Cu(I) is also produced when CctR is incubated with Cu(II). Group 0: 1 mM BCA + 100 μM $CuSO_4$, no protein. Group 1: 10 μM protein + 1 mM BCA, no $CuSO_4$. Group 2: 10 μM CctR protein + 1 mM BCA + 100 μM $CuSO_4$.

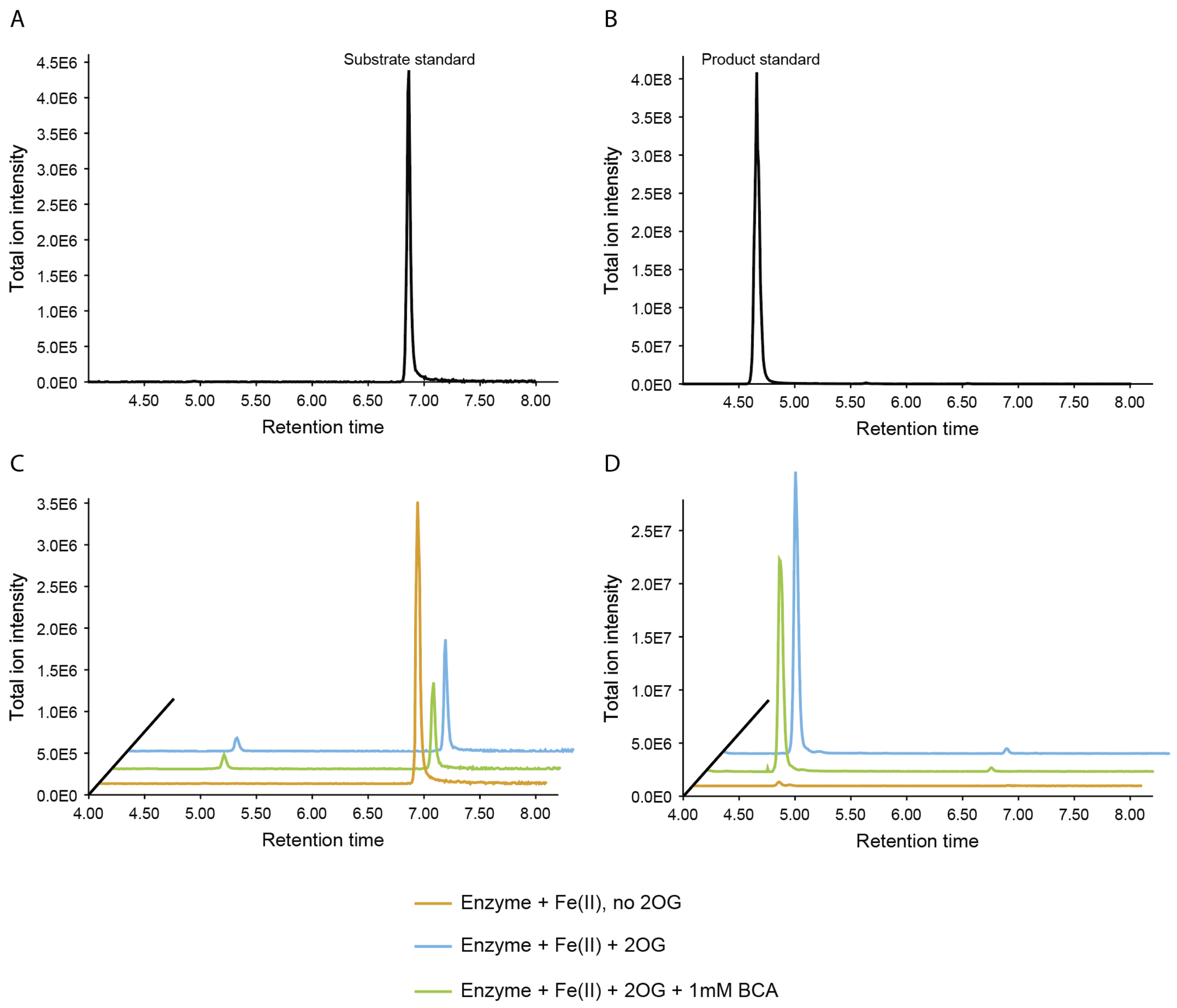

Figure S12. Activity of a 2OGD hydroxylase enzyme in the presence and absence of BCA. (**A**, **B**) Extracted ion chromatograms of the substrate and product standards, respectively. (**C**, **D**) Extracted ion chromatograms showing substrate consumption (**C**) and product formation (**D**) after the enzyme assay. The enzyme's activity was not affected by the addition of BCA.

A

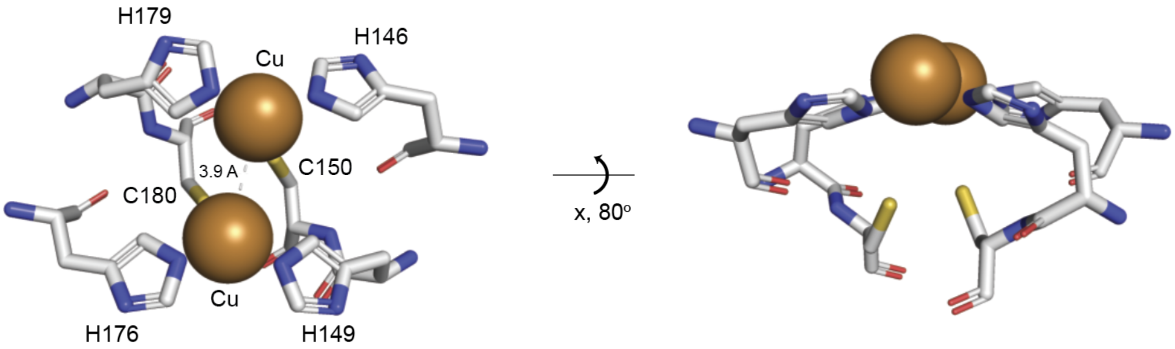

B

	Batch 1			
	Replicate 1	Replicate 2	Replicate 3	Mean (sd)
Molar ratio of Cu:CctR(monomeric count)	2.0268	2.0174	2.0231	2.0224 (0.0047)

	Batch 2			
	Replicate 1	Replicate 2	Replicate 3	Mean (sd)
Molar ratio of Cu:CctR(monomeric count)	2.3103	2.1730	2.2415	2.2409 (0.0686)

C D

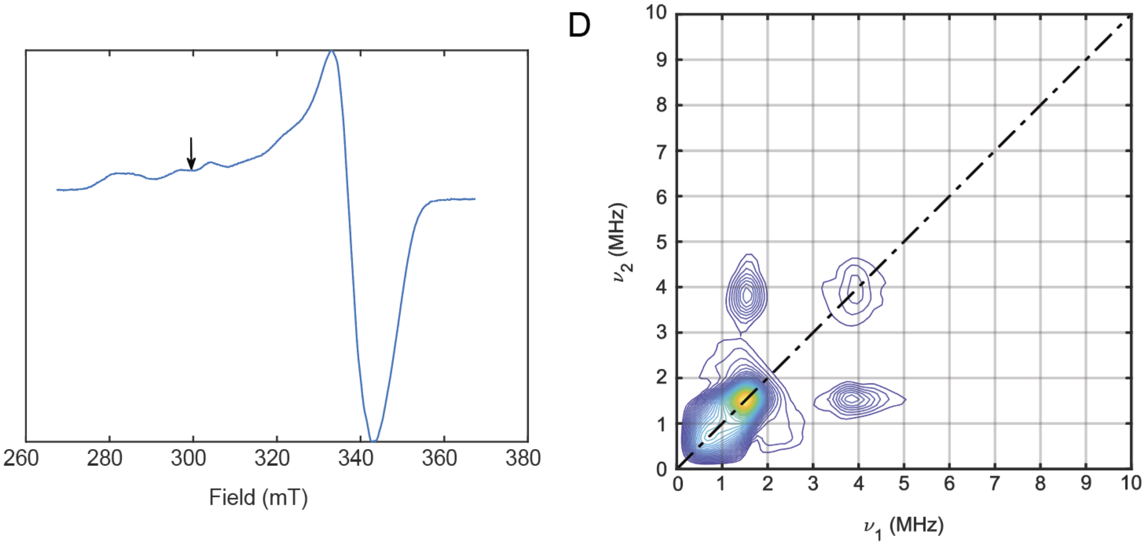

Figure S13. Structural and spectroscopic characterization of copper binding in CctR. (**A**) AlphaFold-predicted structural model of the CctR dimer, showing two copper atoms per monomer. Copper atoms were automatically placed by AlphaFold at the center of the HxxHC$(x)_n$HxxHC motif. (**B**) Quantification of the molar ratio of copper to protein molecule (monomeric count) in

copper-bound CctR protein. In batch 1, the protein was saturated with 4 equivalent Cu^{2+} for copper binding. In batch 2, the protein was saturated with 5 equivalent Cu^{2+} for copper binding. After 2-hour binding, excess unbound copper was then washed away by 3-day dialysis. Copper content was quantified by ICP-MS, while protein concentration was measured by CBQCA Plus Protein Quantitation Kit (Thermo Fisher). (**C**) EPR spectrum of Cu-bound CctR at 139 K showing the characteristic hyperfine splitting of the Cu^{2+} nucleus. Arrow indicates the position in the spectrum at which HYSCORE data were acquired. (**D**) (+,+) HYSCORE[17] spectrum of the same sample at 20 K. The spectra exhibit single- and double-quantum peaks at 1.48 and 3.85 MHz respectively, demonstrating that at least one nitrogen atom is coupled with the Cu^{2+} center.

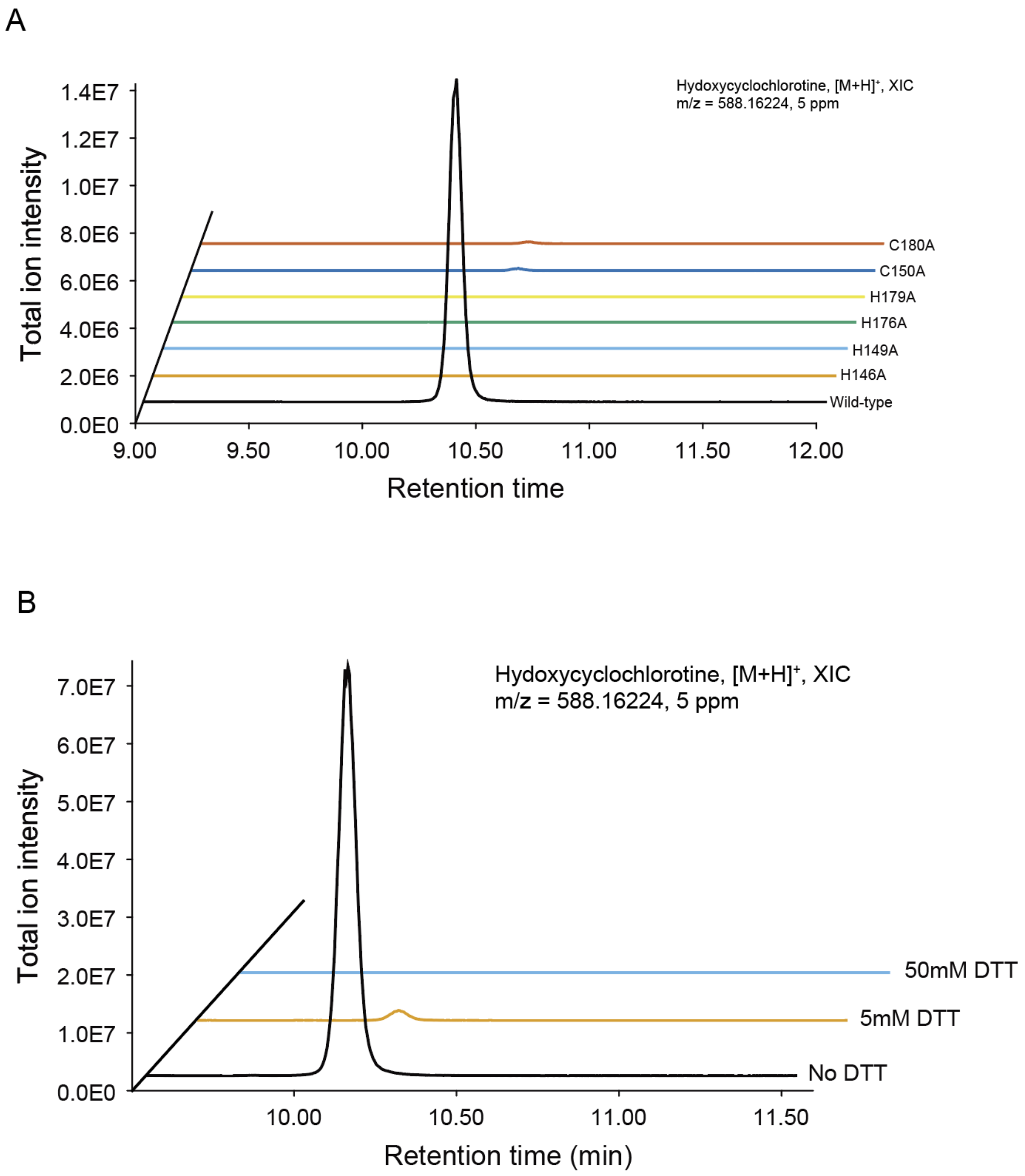

Figure S14. Disruption of the HxxHC(x)$_n$HxxHC motif or reductive treatment impairs CctR activity. (**A**) Enzymatic activities of CctR mutants in which the histidine and cysteine residues of the HxxHC(x)$_n$HxxHC motif are mutated to alanine. (**B**) Enzymatic activities of CctR following reduction with DTT. All assays were conducted using 5 µM protein, 10 µM Cu(II), and 10 mM ascorbate.

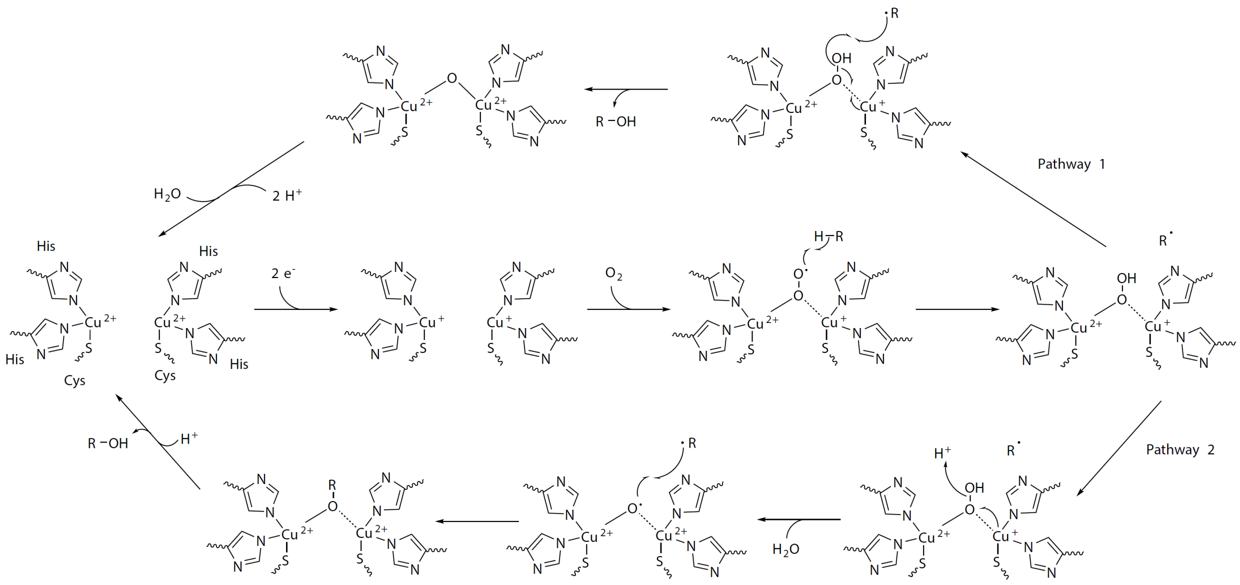

Figure S15. Proposed reaction mechanisms for the hydroxylation reaction catalyzed by CctR. Following hydrogen atom abstraction, two possible pathways are proposed for hydroxylation of the resulting carbon radical.

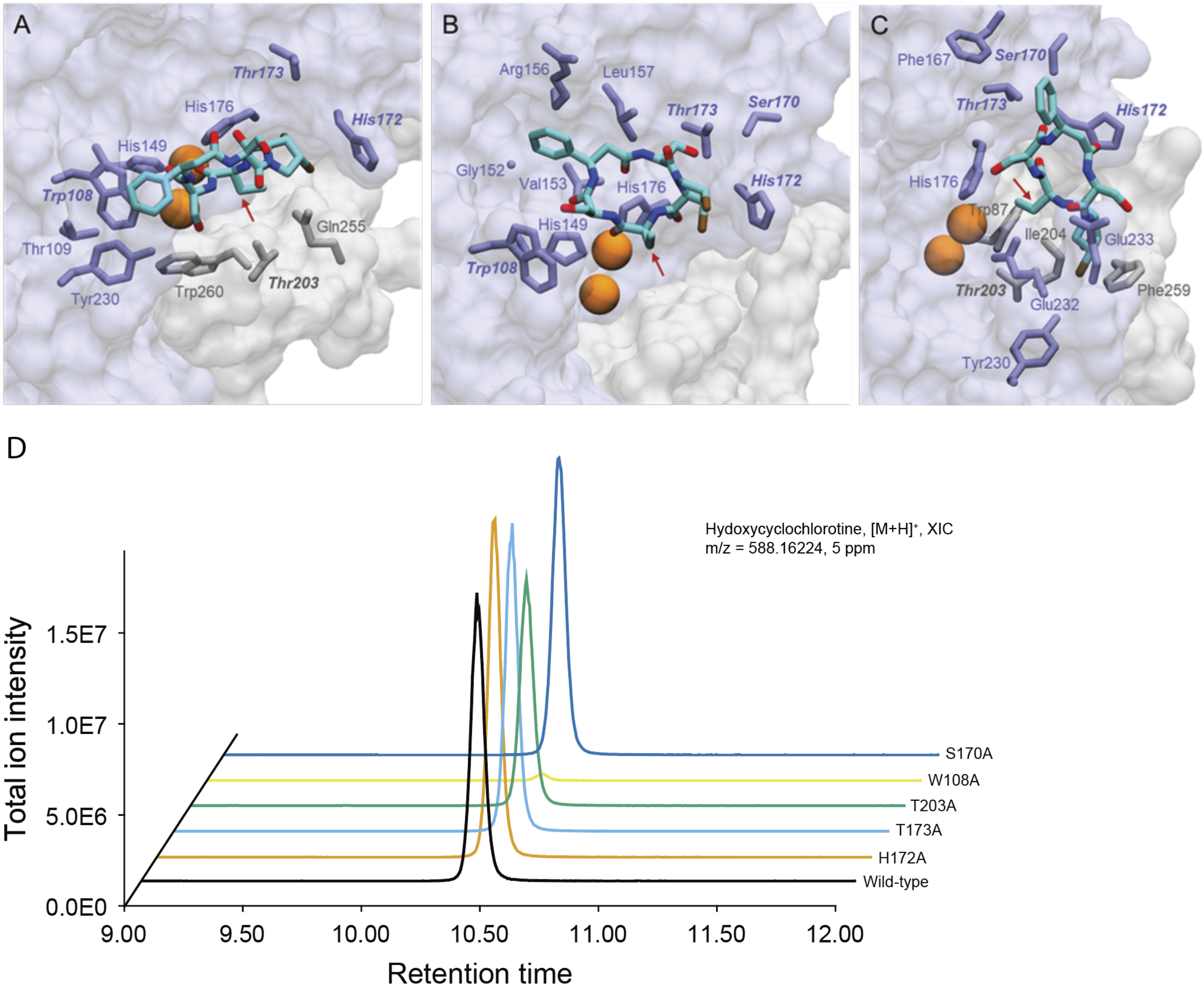

Figure S16. MD simulations and mutagenesis identify key CctR residues involved in cyclochlorotine binding. (**A-C**) Representative conformations of the CctR-cyclochlorotine complex captured by MD simulations. CctR is shown in a surface representation, with its two monomers colored in ice blue and silver, respectively. Copper ions are depicted as orange spheres. C_α atoms and side chains of CctR residues interacting with cyclochlorotine are labeled. The amino acids subjected to mutagenesis experiments are shown in bold italic font. Dark red arrows highlight the β-carbon (C_β) in the 2-aminobutyrate residue of cyclochlorotine. The distances between C_β and copper ions are: (**A**) 4.4 Å, (**B**) 4.6 Å, and (**C**) 4.7 Å, respectively. (**D**) *In vitro* assay showing the activities of CctR mutants from alanine substitutions of selected residues identified in MD simulations. Except for W108A showing significantly reduced activity, all other mutant enzymes retained similar catalytic activity to wild-type enzyme.

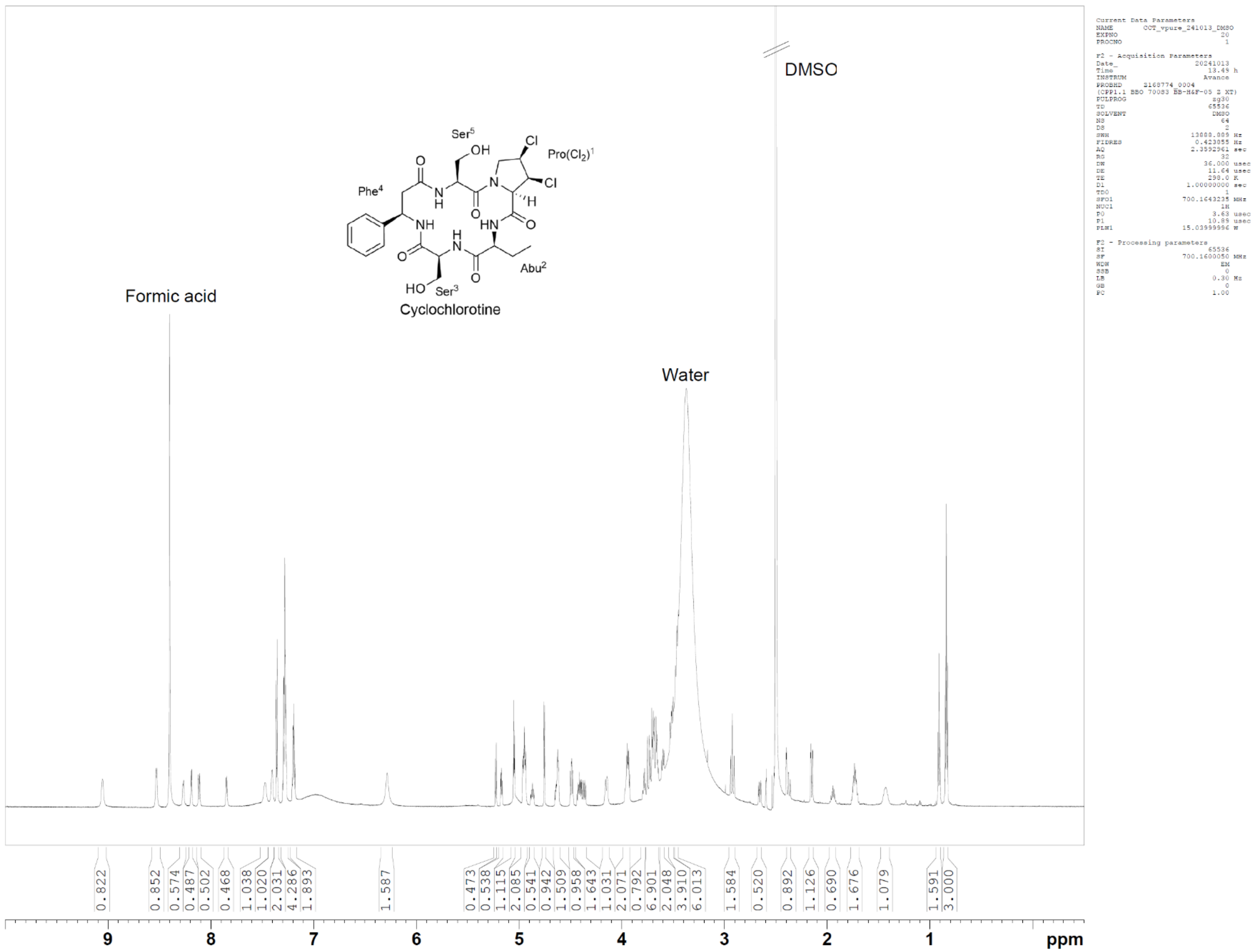

Figure S17. ^{1}H NMR spectrum of cyclochlorotine in DMSO-d_6 (298 K, 700 MHz).

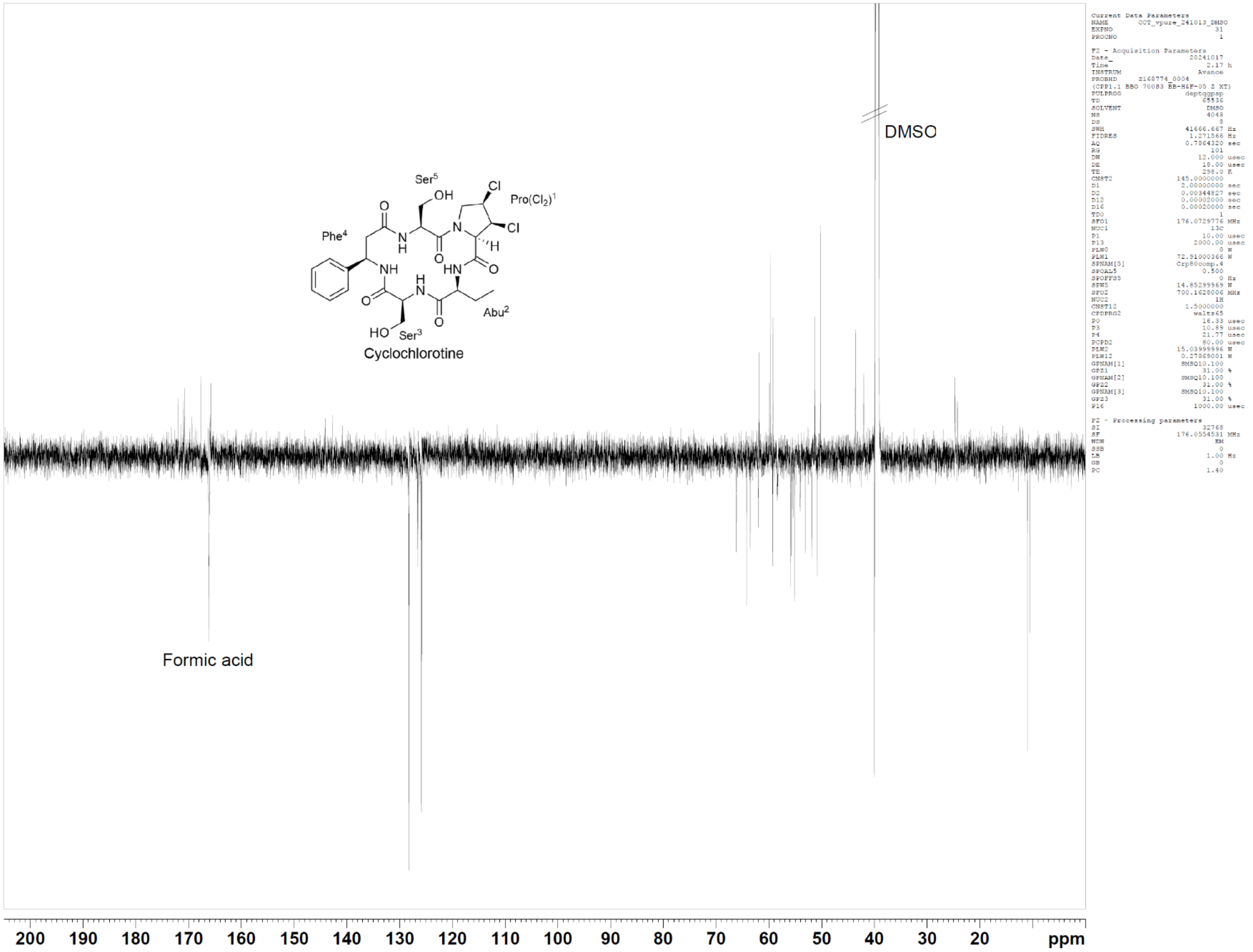

Figure S18. ^{13}C DEPTQ spectrum of cyclochlorotine in DMSO-d_6 (298 K, 700 MHz).

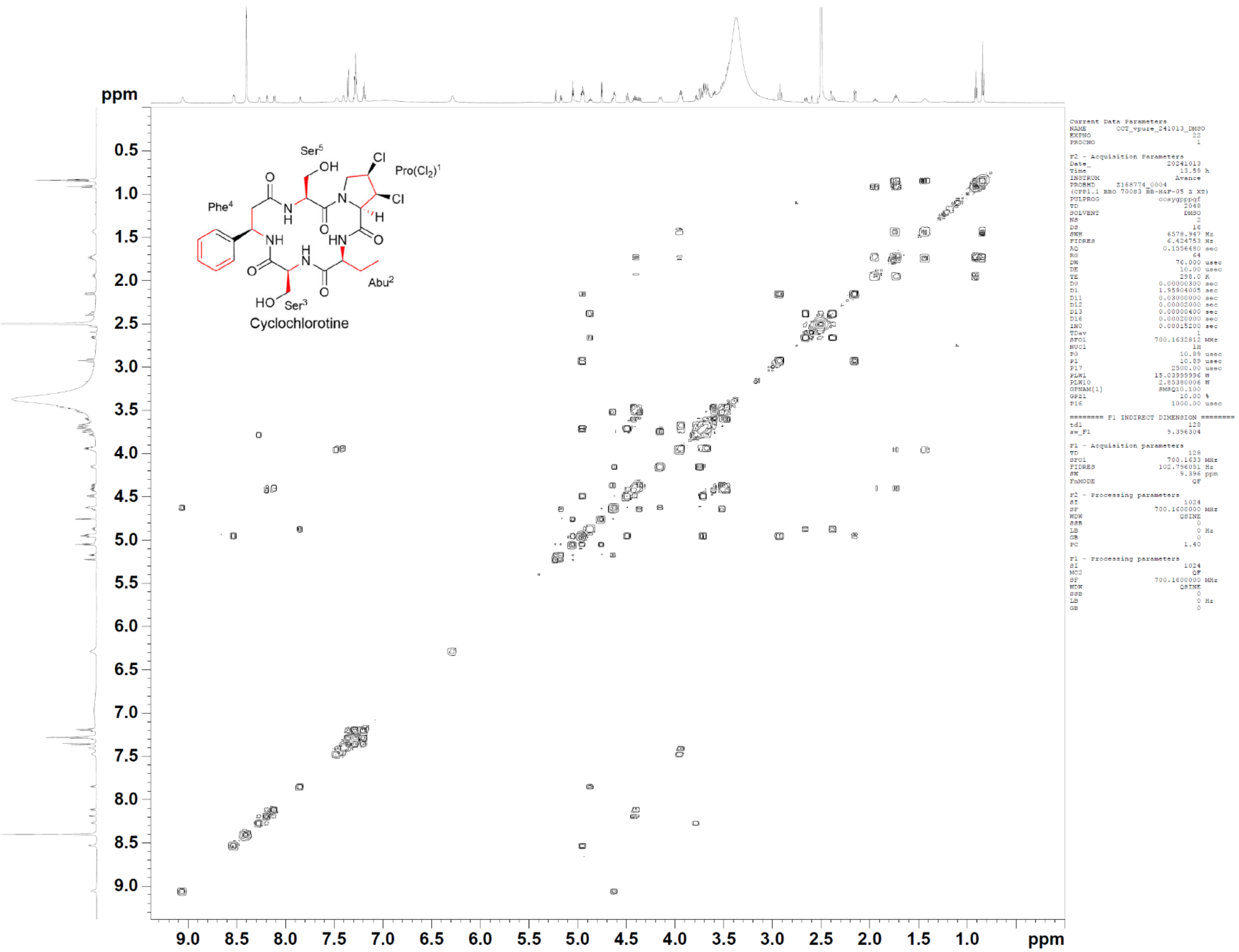

Figure S19. COSY spectrum of cyclochlorotine in DMSO-d$_6$ (298 K, 700 MHz).

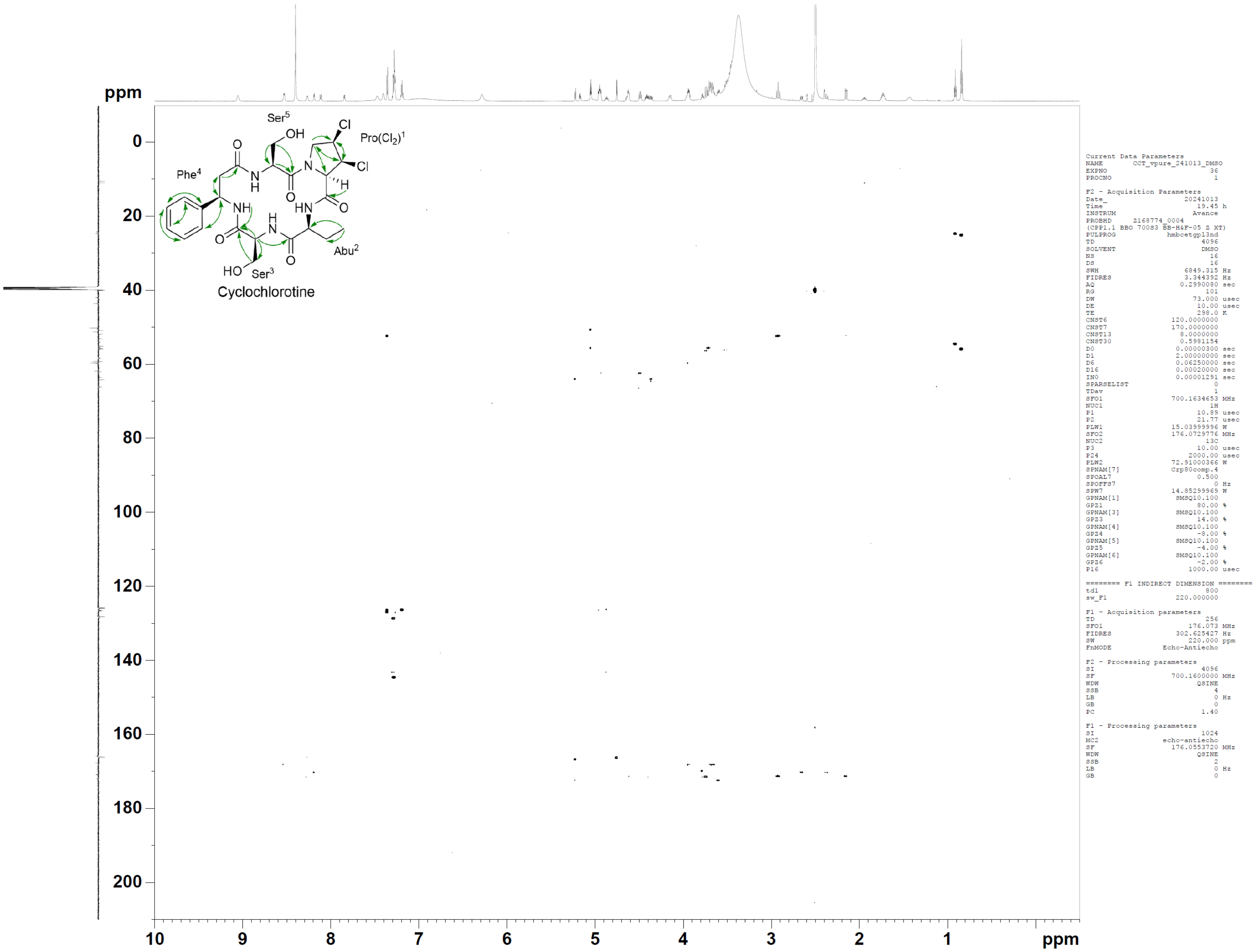

Figure S20. HMBC spectrum of cyclochlorotine in DMSO-d$_6$ (298 K, 700 MHz).

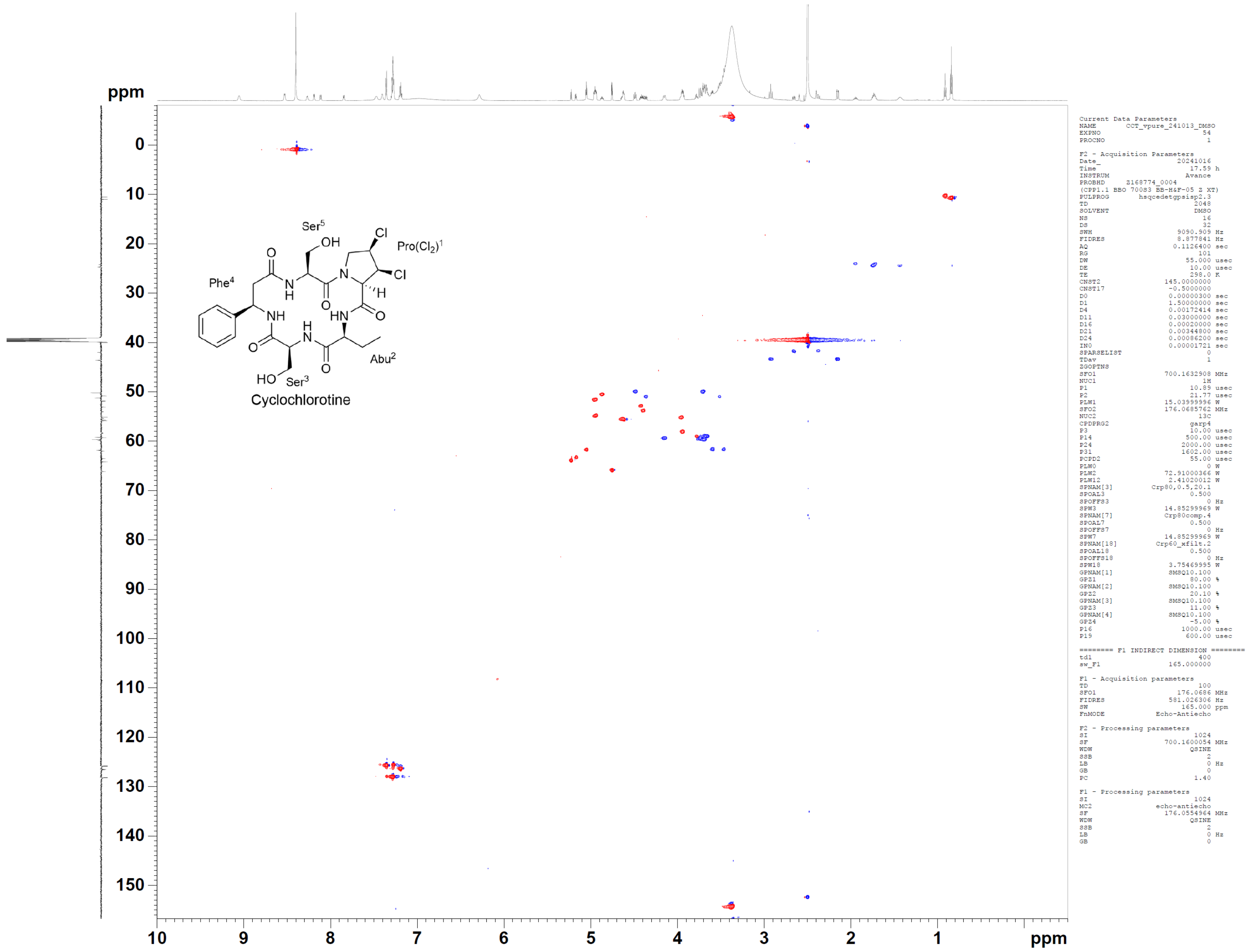

Figure S21. HSQC-DEPT spectrum of cyclochlorotine in DMSO-d_6 (298 K, 700 MHz).

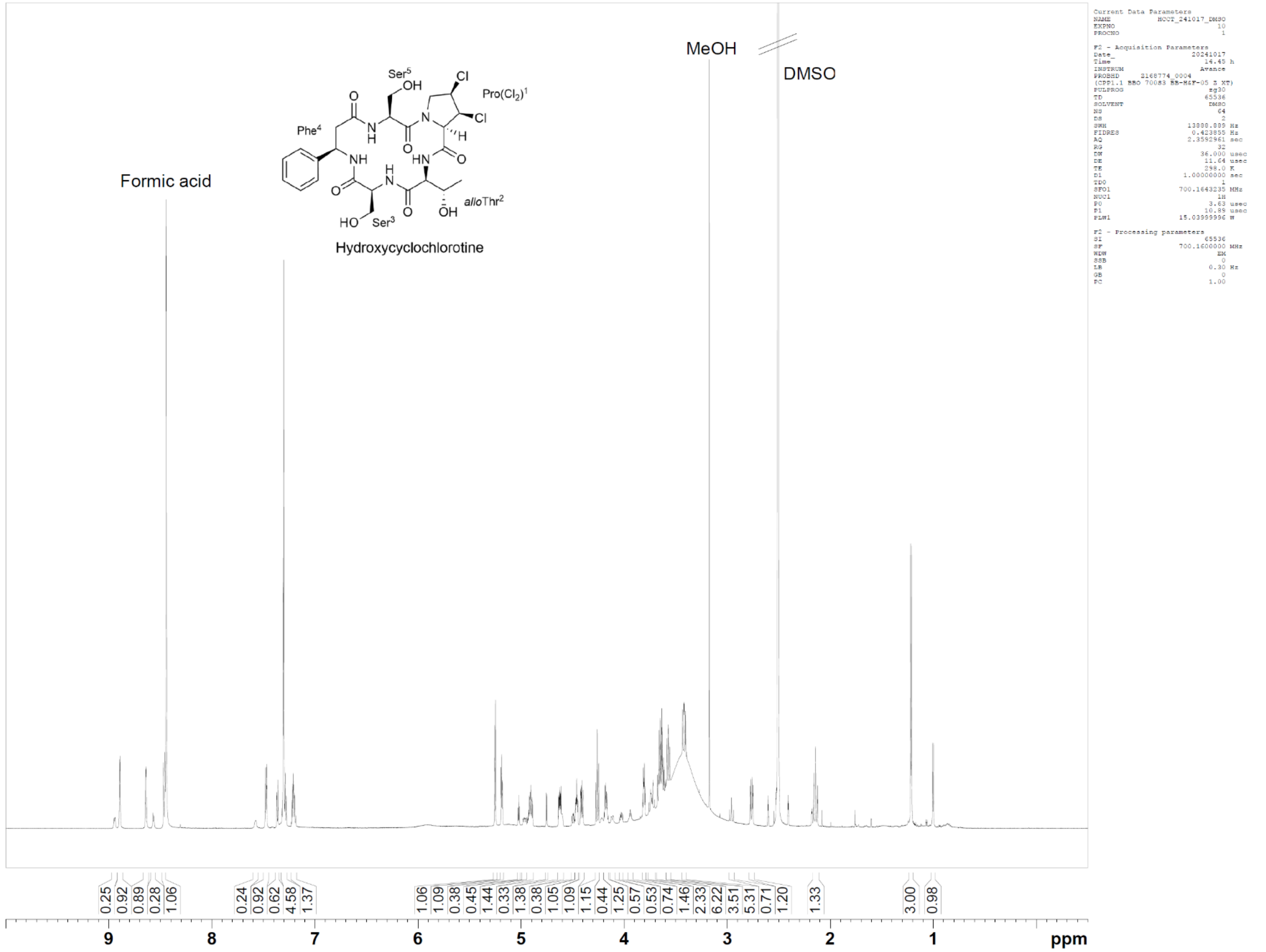

Figure S22. ^{1}H NMR spectrum of hydroxycyclochlorotine in DMSO-d_6 (298 K, 700 MHz).

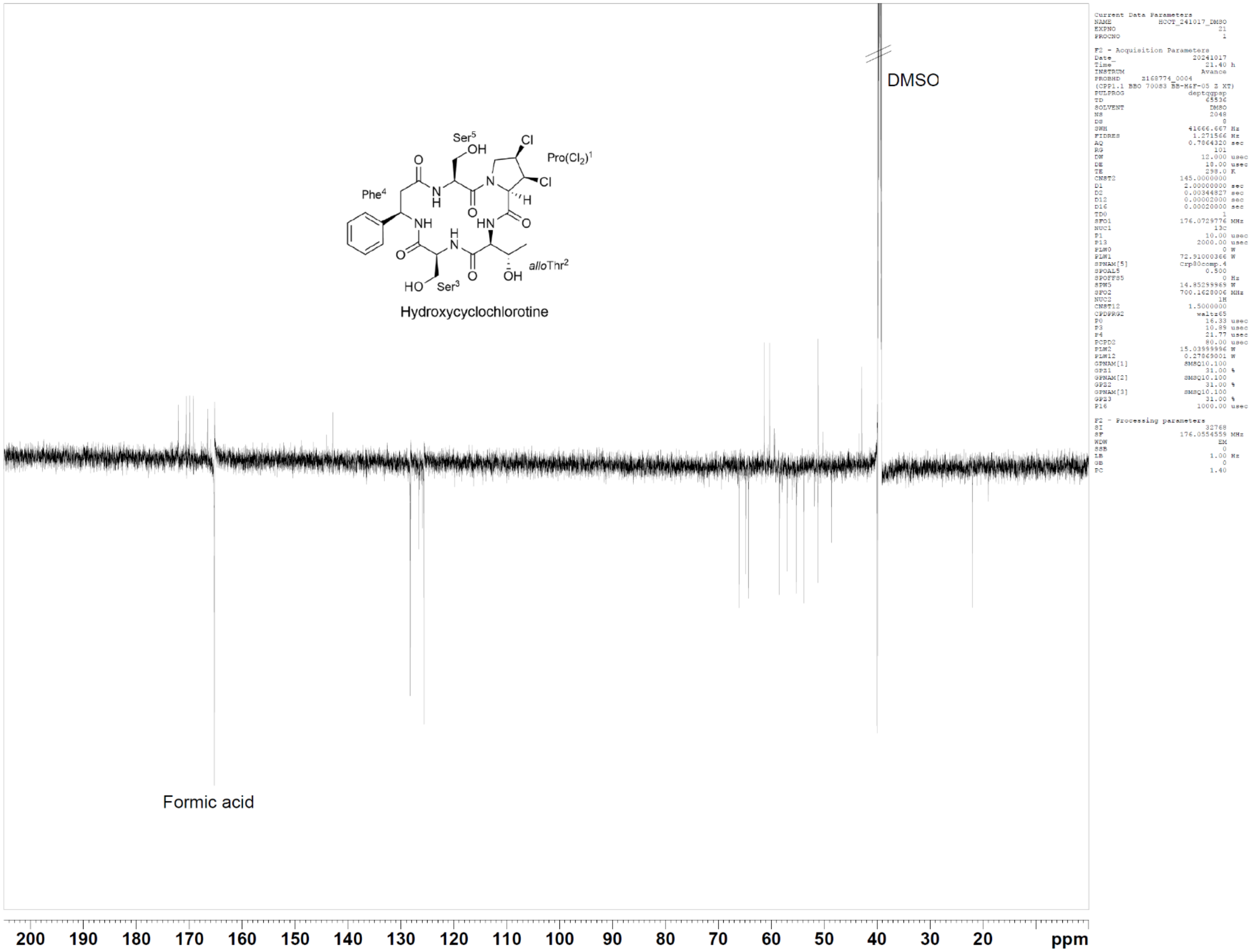

Figure S23. ^{13}C DEPTQ spectrum of hydroxycyclochlorotine in DMSO-d$_6$ (298 K, 700 MHz).

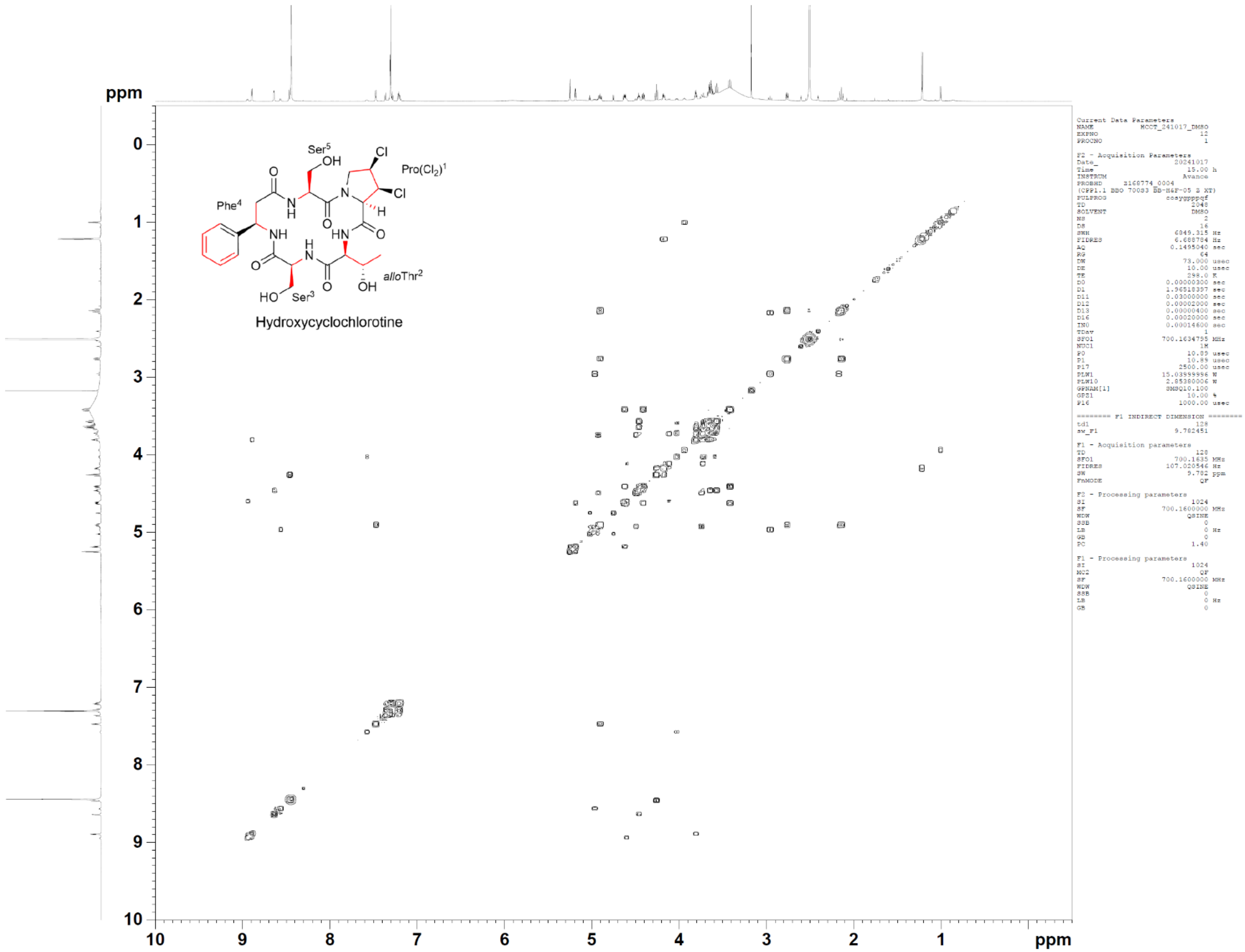

Figure S24. COSY spectrum of hydroxycyclochlorotine in DMSO-d$_6$ (298 K, 700 MHz).

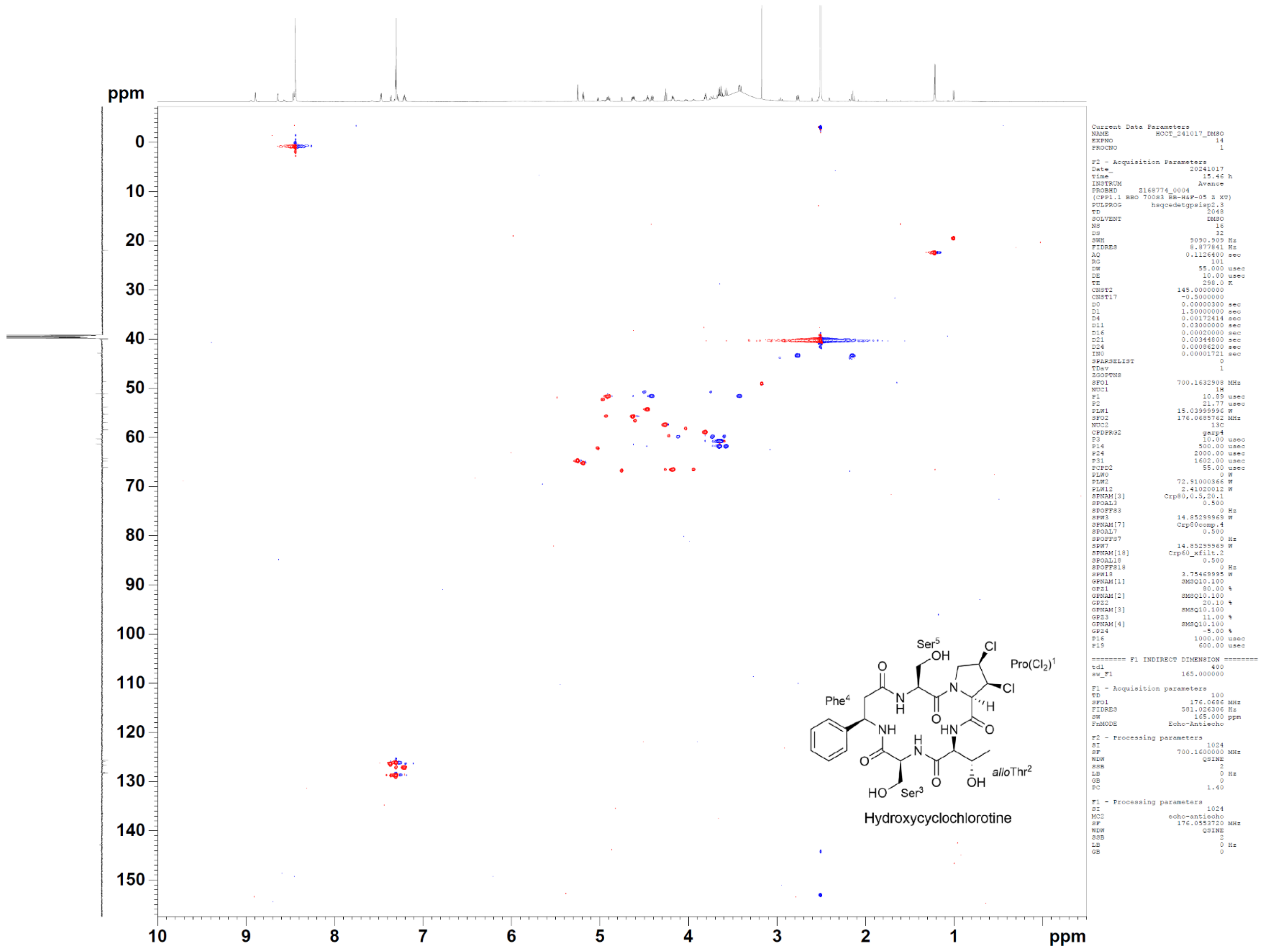

Figure S25. HSQC-DEPT spectrum of hydroxycyclochlorotine in DMSO-d$_6$ (298 K, 700 MHz).

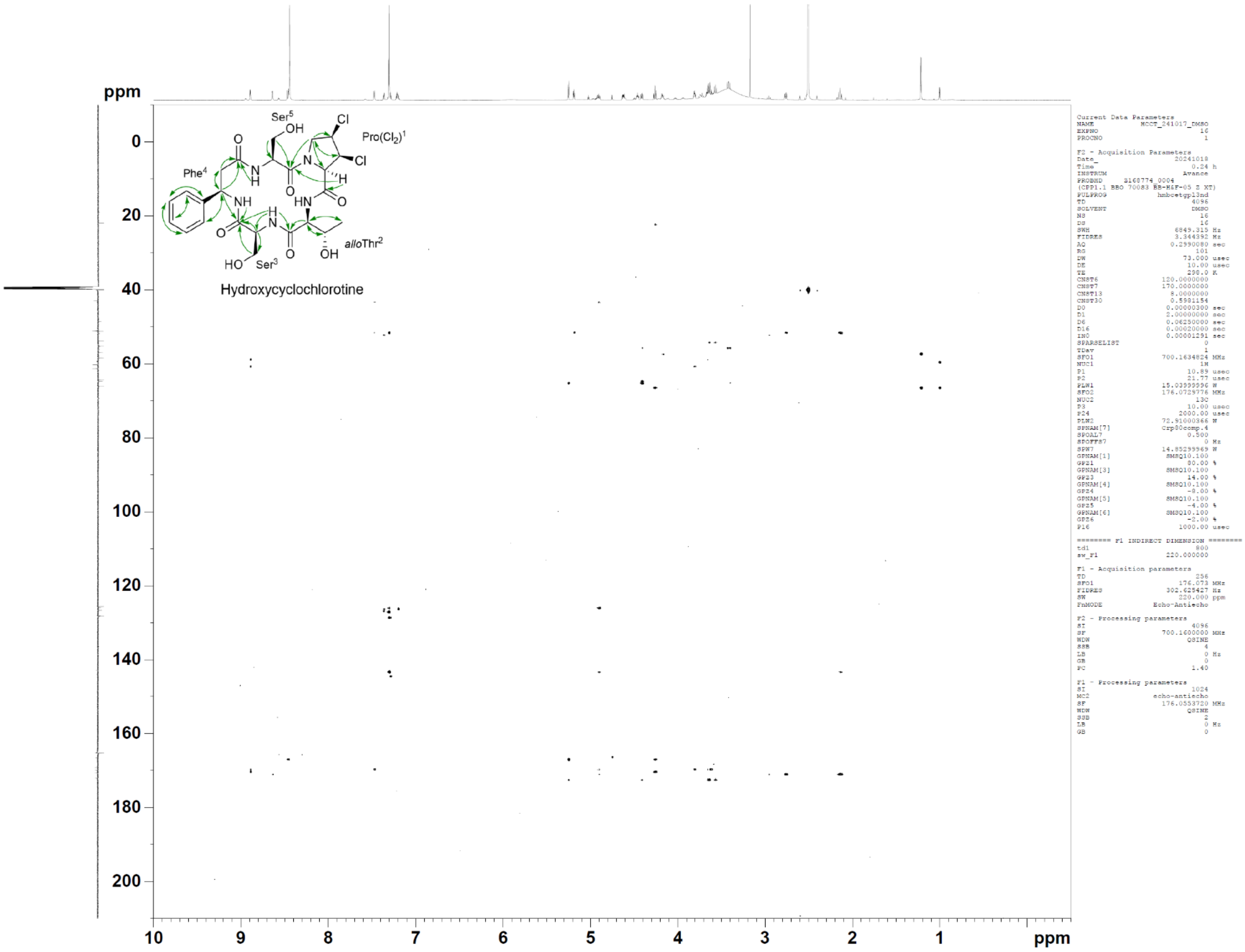

Figure S26. HMBC spectrum of hydroxycyclochlorotine in DMSO-d_6 (298 K, 700 MHz).

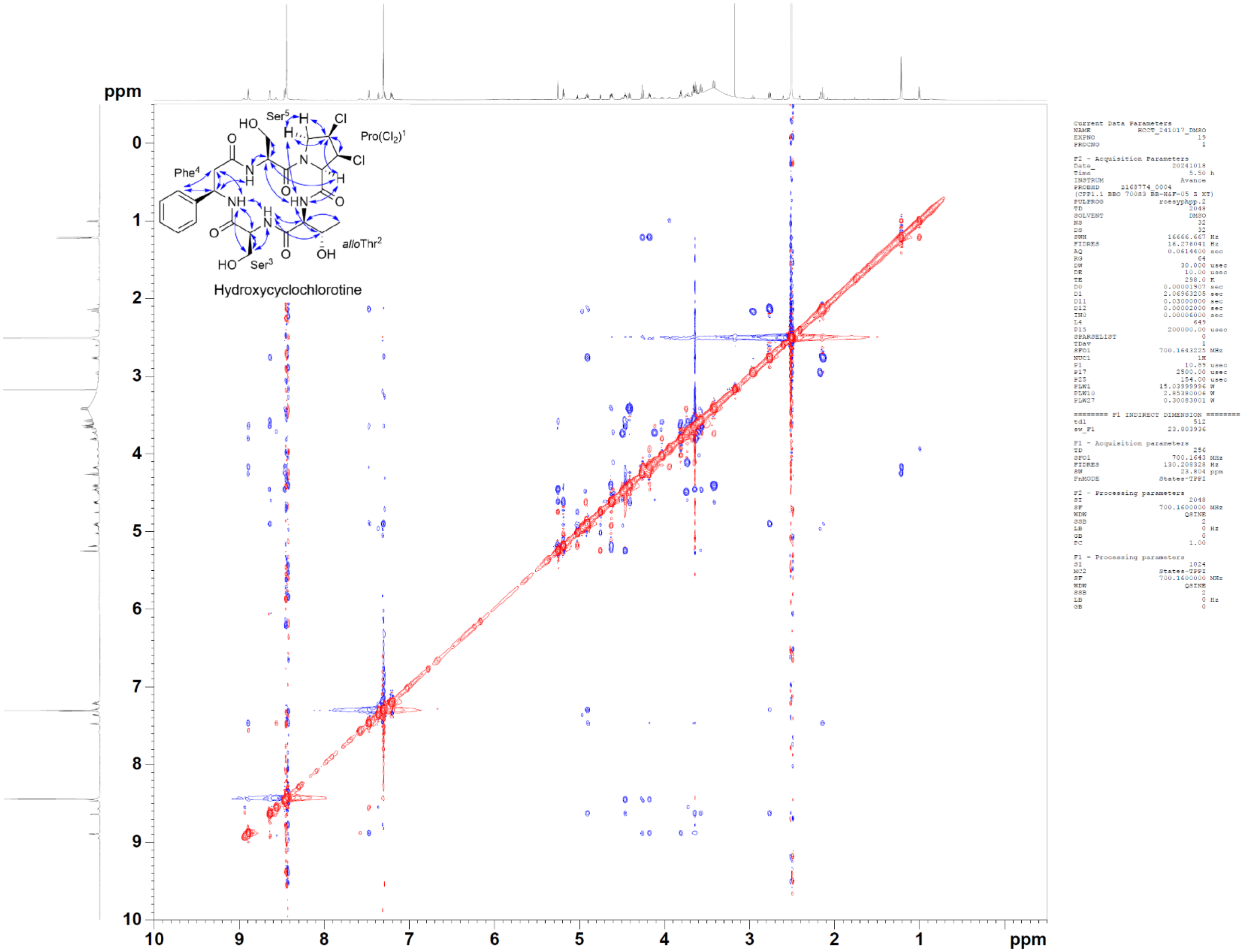

Figure S27. ROESY spectrum of hydroxycyclochlorotine in DMSO-d$_6$ (298 K, 700 MHz).

Supplementary Tables

Table S1. Abundance of remaining substrate and product in the assay with/without reducing agent. The LC/MS chromatograph peak area of the cyclochlorotine and hydroxycyclochlorotine are shown as the compound abundance in the table below. In order to compare the conversion rate at the end of reaction, excessive substrate was used in the assay.

	Remaining substrate (cyclochlorotine)	Product (hydroxycyclochlorotine)	Ratio of product/remaining substrate
Cu(II), no reducing agent, replicate 1	517,571,450.22	6,689,821.26	1.29%
Cu(II), no reducing agent, replicate 2	541,429,980.86	6,895,295.99	1.27%
Cu(II) + NADH, replicate 1	461,401,519.30	23,150,150.09	5.02%
Cu(II) + NADH, replicate 2	464,595,438.44	26,923,114.32	5.79%
Cu(II) + NADPH, replicate 1	469,436,498.42	33,092,274.69	7.05%
Cu(II) + NADPH, replicate 2	450,538,386.94	37,028,549.51	8.22%
Cu(II) + Ascorbate, replicate 1	530,112,533.12	120,844,246.06	22.80%
Cu(II) + Ascorbate, replicate 2	529,366,163.16	120,201,014.49	22.71%

Table S2. ^{1}H and ^{13}C NMR data of cyclochlorotine and hydroxycyclochlorotine in DMSO-d_6 (700 MHz). Both compounds are present in DMSO as two distinct conformers with differing chemical shifts. Where possible, NMR data of both conformers were assigned separately. Peaks that were not found are marked as not detected (n.d.).

		Cyclochlorotine (**1**)				Hydroxycyclochlorotine (**2**)			
		δ_H, multiplicity(J)		δ_C		δ_H, multiplicity(J)		δ_C	
		major	minor	major	minor	major	minor	major	minor
Pro(Cl_2)[1]	C=O			165.8	166.2			166.5	165.9
	α	4.75, d (4.7)	5.22, d (5.9)	66.1	64.2	5.25, d (5.50)	4.75, d (4.7)	64.3	66.3
	β	5.05, dd (4.7, 4.30)	5.17, dd (5.9, 4.7)	62.0	63.5	5.18, dd (5.4, 4.2)	5.02, t (4.7, 4.3)	64.8	61.7
	γ	4.95, m	4.64, m	55.1	55.7	4.62, ddd (9.2, 6.6, 4.2)	4.92, m	55.3	55.2
	ε	3.71, m	3.51, dd (11.7, 8.7)	50.2	51.3	3.41, dd (11.2, 9.3)	4.49, dd (9.7, 7.0)	51.2	50.3
	NH	4.49, dd (9.7, 7.2)	4.36, dd (11.7, 6.6)			4.40, dd (11.2, 6.6)	3.74, m		
Abu (**1**)[2] /*allo*Thr(**2**)[2]	C=O			171.9	171.0			169.9	n.d.
	α	3.95, m	4.40, m	55.4	54.1	4.26, t (9.5, 9.5)	4.21, m	56.9	59.1
	β	1.43, m	1.73, m	24.7	24.2	4.17, m	3.93, t (6.6, 6.6)	66.1	66.1
		1.73, m	1.94, m	24.7	24.2				
	γ	0.84, t (7.4, 7.4)	0.91, t (7.4, 7.4)	11.0	10.5	1.21, d (6.1)	1.00, m	21.9	19.0
	NH	7.47, m	8.12, d (9.3)			8.46, d (9.5)	7.33, m		
Ser[3]	C=O			167.7	169.3			169.2	n.d.
	α	3.93, m	3.78, dt (6.6, 6.6)	58.4	59.3	3.80, dt (6.8, 4.5, 4.5)	4.6, m	58.4	56.1
	β	3.66, m	3.69, m	59.2	59.9	3.63, m	4.11, br dd (11.8, 3.3)	60.3	59.5
						3.66, m	3.72, m		
	NH	7.41, br d (3.8)	8.27, br d (5.5			8.89, d (4.5)	8.94, br d (7.0)		
Phe[4]	C=O			170.8	169.8			170.5	n.d.
	α	2.15, dd (12.6, 2.1)	2.38, m	43.6	42.0	2.13, dd (12.5, 12.9)	2.95, br dd (12.6, 12.6)	42.8	43.4
		2.92, t (12.6, 12.6)	2.66, dd (13.9, 4.6)			2.76, dd (12.9, 4.7)	2.16, m		
	β	4.95, m	4.87, m	51.9	50.8	4.90, m	4.96, ddd (12.6, 7.3, 2.1)	51.1	51.8
	γ			144.1	144.1			142.9	n.d.
	δ	7.37, m	7.35, u	125.9	125.8	7.30, m	7.36, m	125.6	125.9
	ε	7.30, m	7.28, m	128.2	128.2	7.30, m	7.35, m	128.2	128.2
	ζ	7.20, m	7.19, m	126.5	126.6	7.20, m	7.30, m	126.6	126.6
	NH	8.53, br d (6.6)	7.85, br d (6.6)			7.47, d (6.6)	8.56, br d (7.2)		
Ser[5]	C=O			171.9	171.0			172.1	n.d.
	α	4.62, br dd (6.9, 3.7)	4.42, m	55.9	53.1	4.46, ddd (9.0, 5.7, 3.5)	4.02, ddd (9.9, 6.3, 5.2)	53.8	57.7
	β	3.74, br d (12.6)	3.46, dd (10.0, 10.0)	59.7	61.8	3.56, m	3.59, m	61.3	59.3
		4.15, br dd (12.6, 3.0)	3.60, dd (10.0, 5.6)			3.64, m	3.71, m		
	NH	9.05, m	8.19, d (4.9)			8.63, d (3.0)	7.57, m		

Table S3. sgRNA sequences used for CRISPR/Cas9-mediated gene deletion in *T. islandicus*

	Sequence
*cctP2*_sgRNA_1	CATGGTGACAACTTTCTCAC
*cctP2*_sgRNA_2	ACGCTCCAAAACGTTCCCCT
*cctP2*_sgRNA_3	TGACACGATAAGAAACGGGG
*cctO*_sgRNA_1	TTCTCAAATTCGTCAATCTG
*cctO*_sgRNA_2	TCGTTTGTTGCGGCGTACGT
*cctO*_sgRNA_3	GTCCACATTGTCGACAGGAA
*cctR*_sgRNA_1	GACTCATCAGGGTCAAGAGT
*cctR*_sgRNA_2	CTTCGGCGTACGACTCATCA
*cctR*_sgRNA_3	TCTTCGGCGTACGACTCATC

Table S4. Amino acid sequences of DUF3328 proteins characterized in this study

	Protein sequence
CctO	MPLYSQIRQSEDAFSEPTYAATNELINDNENACPHCRQERSESWFLKGGRSIVYVSLT FFVVSIGLNFILAILLYSKFHDILRQYTWLLSGKYTTDGIPVPIFLQKPPEENRRHVDQ CIEELRMGLMCHGDMTPLLITKKRDGASGFKADMNTHYMCRNFTKLQEWTMSHG VEHWELGDGRGPHEHGRR
CctP2	MEGKTSRYQDEAHDSAGSFNEETEGLMSGLHRSTKKRKLSSIVKLATPFLIVSFILNI VQLAYITVRRPECYSLYAKLKEHEITVPFRYATEYSDDEHTHEEKDALWNAIDISEGF VAISNDESDRLGLPRSKTFPWDANKGIYVSHGHHALHCTVLLHAYTYDAHQGKKPL VSYHHIEHCLDLLRQDIMCYANDVMDYTPDHGDNFLTGEGQQRKCRDWNKLSAW VKERSACYKTINITRAGEDHGVAHQLDRYTYCPPGSPYEPLIKAFKDLGRVNTGNLA ADGFHELTPEELAAEAQAVAEHNKQILADEG
CctR	MEEELEPLNRPTLDPDESYAEEKIYGSSHREPNSRIRVFVSLLILSNTISFGLLGWIGLS STQASLAIPEDYAIPPRIATQYKRFWWTTEYSSKNQSQQDELWNSIVWTYGMIGVDH EWSKSQHWPDTMSLPQDKTKAVYLLQAYHEIHCLGVLRRLMSQSLAGVDFSESEHT HAHIAHCFDSLLQSTICRADSTPLYTFGGTIVGSGQQHECRDWNALRDYATQNSACY TEESGFGGQCSDGDGLVPATPMETQQDGFWL

References

[1] R. Schmid, S. Heuckeroth, A. Korf, A. Smirnov, O. Myers, T. S. Dyrlund, R. Bushuiev, K. J. Murray, N. Hoffmann, M. Lu, A. Sarvepalli, Z. Zhang, M. Fleischauer, K. Duhrkop, M. Wesner, S. J. Hoogstra, E. Rudt, O. Mokshyna, C. Brungs, K. Ponomarov, L. Mutabdzija, T. Damiani, C. J. Pudney, M. Earll, P. O. Helmer, T. R. Fallon, T. Schulze, A. Rivas-Ubach, A. Bilbao, H. Richter, L. F. Nothias, M. Wang, M. Oresic, J. K. Weng, S. Bocker, A. Jeibmann, H. Hayen, U. Karst, P. C. Dorrestein, D. Petras, X. Du, T. Pluskal, *Nat Biotechnol* **2023**, *41*, 447-449.

[2] K. Mizutani, Y. Hirasawa, Y. Sugita-Konishi, N. Mochizuki, H. Morita, *J Nat Prod* **2008**, *71*, 1297-1300.

[3] J. T. Robinson, H. Thorvaldsdottir, W. Winckler, M. Guttman, E. S. Lander, G. Getz, J. P. Mesirov, *Nat Biotechnol* **2011**, *29*, 24-26.

[4] S. Salazar-Cerezo, R. S. Kun, R. P. de Vries, S. Garrigues, *Enzyme Microb Technol* **2020**, *133*, 109463.

[5] J. P. Concordet, M. Haeussler, *Nucleic Acids Res* **2018**, *46*, W242-W245.

[6] T. Schafhauser, N. Kirchner, A. Kulik, M. M. Huijbers, L. Flor, T. Caradec, D. P. Fewer, H. Gross, P. Jacques, L. Jahn, J. Jokela, V. Leclere, J. Ludwig-Muller, K. Sivonen, W. J. van Berkel, T. Weber, W. Wohlleben, K. H. van Pee, *Environ Microbiol* **2016**, *18*, 3728-3741.

[7] M. T. Marty, A. J. Baldwin, E. G. Marklund, G. K. Hochberg, J. L. Benesch, C. V. Robinson, *Anal Chem* **2015**, *87*, 4370-4376.

[8] M. J. Abraham, T. Murtola, R. Schulz, S. Páll, J. C. Smith, B. Hess, E. Lindahl, *SoftwareX* **2015**, *1-2*, 19-25.

[9] J. Huang, S. Rauscher, G. Nawrocki, T. Ran, M. Feig, B. L. de Groot, H. Grubmüller, A. D. MacKerell, Jr., *Nat Methods* **2017**, *14*, 71-73.

[10] K. Vanommeslaeghe, E. Hatcher, C. Acharya, S. Kundu, S. Zhong, J. Shim, E. Darian, O. Guvench, P. Lopes, I. Vorobyov, A. D. Mackerell, Jr., *J Comput Chem* **2010**, *31*, 671-690.

[11] G. Bussi, D. Donadio, M. Parrinello, *J Chem Phys* **2007**, *126*, 014101.

[12] O. Trott, A. J. Olson, *J Comput Chem* **2010**, *31*, 455-461.

[13] M. Bernetti, G. Bussi, *J Chem Phys* **2020**, *153*, 114107.

[14] T. Darden, D. York, L. Pedersen, *The Journal of Chemical Physics* **1993**, *98*, 10089-10092.

[15] B. Hess, H. Bekker, H. J. C. Berendsen, J. G. E. M. Fraaije, *Journal of Computational Chemistry* **1997**, *18*, 1463-1472.

[16] W. Humphrey, A. Dalke, K. Schulten, *Journal of Molecular Graphics* **1996**, *14*, 33-38.

[17] S. Van Doorslaer, in *eMagRes*, pp. 51-70.

[18] L. Fábregas Ibáñez, J. Soetbeer, D. Klose, M. Tinzl, D. Hilvert, G. Jeschke, *J Magn Reson* **2019**, *307*, 106576.

[19] L. Kall, A. Krogh, E. L. Sonnhammer, *J Mol Biol* **2004**, *338*, 1027-1036.

[20] T. J. Wheeler, J. Clements, R. D. Finn, *BMC Bioinformatics* **2014**, *15*, 7.

[21] S. C. Potter, A. Luciani, S. R. Eddy, Y. Park, R. Lopez, R. D. Finn, *Nucleic Acids Res* **2018**, *46*, W200-W204.

[22] J. Mistry, S. Chuguransky, L. Williams, M. Qureshi, G. A. Salazar, E. L. L. Sonnhammer, S. C. E. Tosatto, L. Paladin, S. Raj, L. J. Richardson, R. D. Finn, A. Bateman, *Nucleic Acids Res* **2021**, *49*, D412-D419.
